# Distribution Characteristics and Ecological Risk Assessment of Organophosphate Esters in Surface Soils of China

**DOI:** 10.3390/toxics12090686

**Published:** 2024-09-23

**Authors:** Guorui Zhou, Yizhang Zhang, Ziye Wang, Mingrui Li, Haiming Li, Chen Shen

**Affiliations:** 1 College of Marine and Environmental Sciences, Tianjin University of Science and Technology, Tianjin 300457, China; zhougr2024@163.com; 2 State Key Laboratory of Environmental Criteria and Risk Assessment, Chinese Research Academy of Environmental Sciences, Beijing 100012, China; zhangyz@craes.org.cn (Y.Z.); wzy972953@163.com (Z.W.); limingrui212@mails.ucas.ac.cn (M.L.)

**Keywords:** OPEs, soil, tempo-spatial variations, risk assessment

## Abstract

The chemical flame retardants represented by organophosphate esters (OPEs) are widely used and have a serious impact on the environment. In this study, we collected data on the exposure levels of ten OPEs in Chinese soils in recent years and performed an ecological risk assessment. The results showed that the levels of OPEs varied considerably throughout different regions of China, with high exposure levels in highly urbanized or industrialized areas such as Guangdong Province and Northeast China, where the mean value was >200 ng/g. The content of OPEs in the soil in industrial and commercial areas was significantly higher than in other regions, indicating that the concentration of OPEs in the soil is closely related to local economic development and the degree of industrialization. Meanwhile, the number of studies reporting on OPEs and their exposure concentrations have increased significantly since 2018. Through the ecological risk assessment, it was found that TCP, EHDPP and TEHP pose high ecological risks. Although some OPEs, such as TCIPP, have low ecological risk levels overall, their high exposure concentrations are still worthy of attention. This study details the general status of OPE contamination in Chinese soils, which can serve as a reference for ecological environmental supervision.

## 1. Introduction

Due to large-scale production and the use of various chemicals, the types of new pollutants are increasing, and the scope of environmental pollution is also expanding [1]. Among them, organophosphate esters are important emerging pollutants [2], which have a variety of uses in life. For example, they can be used as herbicides and pesticides in agriculture to reduce the impact of pests and weeds on crops, and they are used in industrial production as flame retardants and plasticizers, among other applications. They are involved in several industries, including building materials, chemicals and textiles [3]. OPEs are phosphoric acid ester derivatives with a common backbone structure of phosphoric acid triglycerides. According to the different functional groups of the side chains, they can be generally categorized into alkyl-substituted phosphate esters (Alkyl-OPEs), chlorinated phosphate esters (Cl-OPEs) and aryl-substituted phosphate esters (Aryl-OPEs) [4]. Halogenated OPEs are mainly used as flame retardants, while non-halogenated OPEs are more commonly used as plasticizers [5].

Brominated flame retardants (BFRs), such as polybrominated diphenyl ethers (PBDEs), were listed as persistent organic pollutants (POPs) by the Stockholm Convention in 2008 due to their persistence, terrestrial food chains and toxicity and bioaccumulation in aquatic environments [6,7]. In recent years, with the restriction of PBDEs, the consumption of OPEs as alternatives to PBDEs has increased significantly on a global scale. The worldwide consumption of OPEs between 2004 and 2011 had risen from 200,000 to 500,000 tons [8,9]; by 2011, the annual production of OPEs in China had reached 100,000 tons, and was expected to grow at an annual rate of 15% [10]. By 2015, the estimated global production of OPEs had reached 680,000 tons. This huge amount of use has resulted in increasing emissions of OPEs into the environment and their widespread detection in air, dust [11,12,13], water [14,15] and sediment [16]. They have also been detected in animals and humans [17]. Due to the persistence, long-distance migration and bioavailability of OPEs, they have quickly attracted widespread attention [18].

In contrast to water and air, the number of domestic and international studies investigating the detection of OPEs in soil is relatively low. Fries [19] conducted a study analyzing the concentration of OPEs in grassland soil in Germany; namely, tris(2-butoxyethyl) phosphate (TBEP), tris(2-chloroisopropyl) phosphate (TCPP) and tris(2-chloroethyl) phosphate (TCEP). The results revealed that the highest concentrations of these compounds reached up to 18.2 ng/g. At an electronic waste recycling facility in Northern Vietnam, the topsoil exhibited elevated concentrations of OPEs, with an average concentration of 724 ng/g [20]. In recent years, a series of studies have demonstrated that OPEs are also frequently detected in soils in China. A survey of farmland soils in 31 provinces in China showed that organophosphorus flame retardants (OPFRs) were widely detected in soil samples [21]. In addition, very high concentrations of OPEs have been observed in soils near manufacturing plants or around various waste recycling areas [22,23]. The average value of topsoil from an open recycling site in Tianjin, China, was found to be 829 ng/g, which was considerably higher than that of farmland soil [24].

When OPEs enter the environment, they not only cause harm to the ecosystem but also pose a threat to human health through the food chain and food web. Toxicological studies have revealed that organophosphates possess significant solubility, rendering them capable of disturbing the nervous system, showcasing neurotoxic effects and inducing reproductive genotoxicity [25]. For instance, tri-n-butyl phosphate (TnBP), triphenyl phosphate (TPHP) and TCEP show neurotoxicity after chronic exposure, and TCEP is also suspected of being carcinogenic [26,27]. Furthermore, pregnant women exposed to OPEs can experience adverse effects including fertilization, implantation and clinical pregnancy [28]. Some countries have implemented legislation to limit the use of OPEs, but at present, there is little research on the toxicity of OPEs to terrestrial organisms, especially soil organisms. Yao et al. conducted experiments examining the effects of varying concentrations of TCEP on wheat seed germination and growth indicators. Their findings revealed that at high concentrations, TCEP significantly inhibited the germination and growth of wheat seeds [29]. Yang et al. conducted toxicity experiments on earthworm Eisenia fetida and found that all five OPEs, TnBP, tricresyl phosphate TCP, tris(1,3-dichloro-2-propyl) phosphate (TDCPP), trimethyl phosphate (TMP) and TCEP, produced different degrees of toxicity to the earthworm Eisenia fetida, with TnBP being the most toxic [30]. 

The issue of emerging pollutants has received widespread attention on a global scale. In May 2022, China introduced the “Action Plan for the Control of Emerging Pollutants” [31], which includes the regulation of OPEs. In recent years, researchers have conducted ecological risk assessments of the status of organophosphate pollution in our environment [32,33,34], but there is a lack of summarization and elaboration on the pollution of our soils. This study investigated ten types of OPEs across thirty-one provincial-level administrative regions in China; conducting a comprehensive analysis of the soil pollution levels of OPEs in China from 2008 to 2022. In this study, we elucidated the relationship between the exposure levels of OPEs in various soils, geographical locations, functional zones and temporal variations. We also conducted source and toxicity analysis and ecological risk assessment.

## 2. Materials and Methods

### 2.1. Data Sourcing and Screening

The data were obtained from the China National Knowledge Infrastructure (CNKI) and Web of Science databases. The search algorithms were (“OPEs” or “OPFRs”), “soil” and “China”. The literature on the concentration and distribution of OPEs in the soil was screened, and sampling point information was extracted. The criteria for adopting literature and materials include a focus on concentrations in the soil environment in China, reliable sampling and detection methods, samples collected from 0 to 20 cm of topsoil and clear position and point information. Articles that do not meet the above criteria were excluded. The samples were taken mainly from urban soil, agricultural soil, industrial area soil, etc. The research and sampling methods used in the course of the study are widely accepted by the scientific community. Soil samples were preserved and transported by national norms. In these studies, the main detection methods for OPEs were gas chromatography–mass spectrometry, high-performance liquid chromatography and tandem mass spectrometry, etc. At the same time, matrix recovery was between 60% and 120% and the calculation of limits of detection (LOD) and limits of quantification (LOQ) were carried out using the standard method of environmental monitoring. Each study included rigorous quality assurance and control. When adopting the data, if the concentration was greater than the Method Detection Limit (MDL), the average concentration of the measurements was used, and if there were multiple samples at different times in a single sampling point, the average of the exposure concentrations at various times in the sampling point was calculated.

### 2.2. Ecological Risk Assessment

The risk assessment of soil OPEs was carried out using risk quotient (RQ) values, which were calculated using the formula in Equation (1):

(1) $$RQ=\frac{{MEC}_{soil}}{{PNEC}_{soil}}$$

where *PNEC_soil_* is the predicted no-effect concentrations of OPEs to the organism and *MEC_soil_* is the measured concentration of OPEs. The maximum potential ecological risks are assessed according to common criteria: 0.01 ≤ RQ < 0.1 indicates a low risk, 0.1 ≤ RQ < 1.0 indicates a medium risk and RQ ≥ 1.0 indicates a high risk [35].

*PNEC_soil_* values for TCEP, TPHP, TDCIPP, TCIPP, TMPP and 2-Ethylhexyl diphenyl phosphate (EHDPP) are based on the European Commission’s report. The *PNEC_soil_* values for TCEP, TCIPP and TDCPP are calculated based on the lowest effect concentration in the terrestrial biome, while the *PNEC_soil_* values for TPHP, EHDPP and TMPP are estimated using the equilibrium partitioning method [35], as shown in Equation (2).

(2) $$\;{PNEC}_{soil}=\frac{K_{soil\text{-}water}}{P_{sed}}\times {PNEC}_{acquatic\text{-}organism}\times 1000$$

where *K_soil-water_* = soil/water partition coefficient = 300 m^3^/m^3^, *P_sed_* = bulk density of wet sediment = 1700 kg/m^3^ and *PNEC_acquatic-organism_* = 0.74 µg/L.

For other OPEs that do not have a European Commission-recommended value, such as TBOEP, TNBP and Tris(2-ethylhexyl) phosphate (TEHP), the corresponding *PNEC_soil_* value is estimated using the ratio of the semi-lethal concentration (LC_50_) of the earthworm to the evaluation factor (f = 1000) [36] (Table S3).

## 3. Results and Discussion

### 3.1. Source of OPEs in Soil

Many daily necessities in human production activities contain OPEs, which are used as flame retardants in various products. For example, foams used to make furniture may contain TDCPP and TCPP. However, these substances escape over time and accumulate in the environment [37]. At the same time, some of the OPEs present in natural bodies of water also enter the soil. Due to the low removal efficiency of OPEs in sewage treatment, the wastewater discharged from sewage treatment plants has become one of the important sources of organophosphates. Moreover, the treated sludge will further penetrate into the soil environment during the landfill process [38]. Not only that, but organophosphate contaminants also include some organophosphorus pesticides [39], which are now widely used in China’s agricultural production, causing the pollution of agricultural soil. OPEs are also ubiquitous in the atmosphere and can be detected not only in outdoor environments, but also in a variety of indoor environments. It is worth noting that these OPEs that exist in the indoor environment will gradually migrate to the external environment through processes of ventilation and dust removal, for example5], achieving long-range transport [40]. They can also bind to airborne particles, greatly enhancing their persistence [6], and eventually entering the soil environment through air–soil exchange. In addition, Takimoto et al. showed that wet and dry deposition may also be a potential method for OPEs to enter the soil [41].

### 3.2. Exposure of Soil to OPEs

#### 3.2.1. OPE Exposure Levels in Soils from Different Regions

According to previous literature data, about 20 types of OPEs are detected in soil, which are divided into three categories: Alkyl-OPEs, Cl-OPEs and Aryl-OPEs. The presence of OPEs has been detected in soils from different regions and is listed in Table 1. Among them, economically developed areas, such as Beijing (range: 21.4–2050 ng/g) [42], Tianjin (range: 37.1–2700 ng/g) [24] and Guangzhou (range: 41–1300 ng/g) [43], have all detected higher concentrations of OPEs, with some sites even exceeding 1000 ng/g. Based on the detection frequency and exposure concentration, ten of these OPEs were screened for the subsequent statistical analysis in this paper: Triethyl phosphate (TEP), TnBP, TBEP, TEHP, TCEP, TDCPP, TCIPP, TCP, TPhP and EHDPP. The basic information of the selected OPEs are summarized in Table S1.

**Figure 1:**
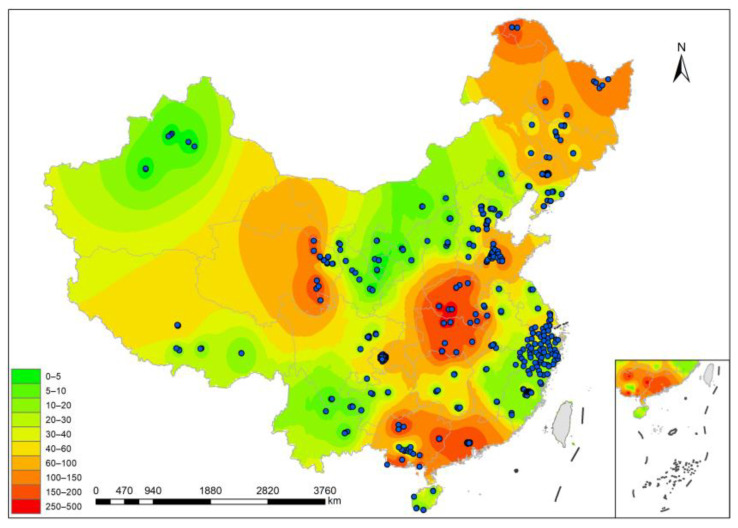
Spatial distribution characteristics of soil organophosphates in China (ng/g dw). The redder the area, the higher the concentration of OPEs in the sample, and the lighter, the lower.

The spatial distribution of OPEs in Chinese soils was analyzed using the Inverse Distance Weight (IDW) method. The total exposure levels of the 10 OPEs in Chinese soils and the sampling points can be seen in Figure 1 (specific information is shown in Table S2). It can be seen that the frequency of surveys on OPEs in China is gradually increasing from the west to the east. The focus of attention is concentrated in central, eastern and southern regions, while there are fewer investigations and studies in remote areas in the west, such as the provinces of Tibet and Xinjiang, meaning that economically developed areas have been the focus of OPE research. Among them, the concentrations of ∑OPEs were significantly higher in the northeastern, central and southern regions than in the western region, which is similar to the findings of Wang et al. [55]. Higher areas include Ningbo (mean: 469 ng/g dw) [48] and the Guangzhou business district (mean: 460 ng/g dw) [43], with high urbanization and industrialization being the main reasons. In contrast, ∑OPEs were lower in the provinces of Gansu (2.4 ng/g dw) and Xinjiang (3.3 ng/g dw) [21], which have low population densities and relatively few industrial activities.

**Figure 2:**
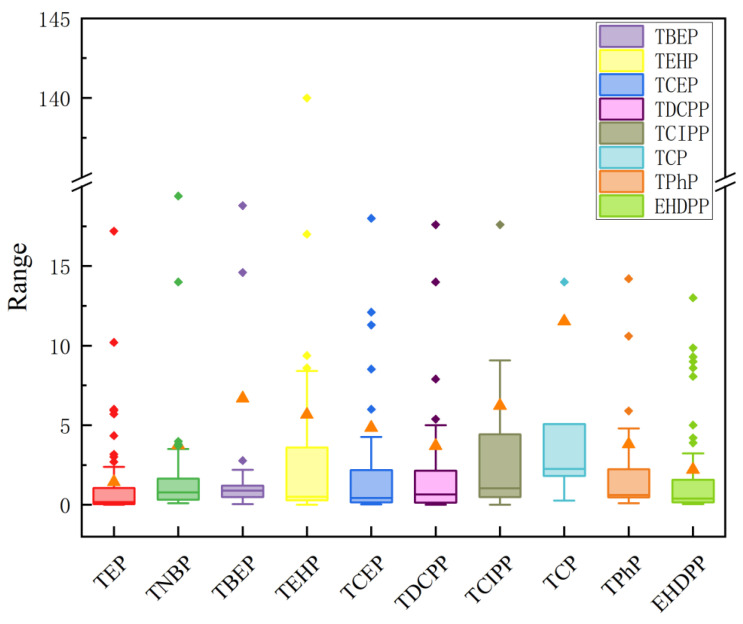
Organophosphate residue concentrations in surface soils in China (ng/g dw). The line in the box represents the median value. Triangles represent average values.

The content of different OPEs in the soils of different regions also varied, with average concentrations of Cl-OPEs > Alkyl-OPEs > Aryl-OPEs, as shown in Figure S1 and Figure 2. Previous studies have shown that Cl-OPEs are the major compounds in Chinese soils, accounting for more than 74.0% of the ∑OPE concentrations [55]. This is similar to the findings of Ji et al. [22] (Table 1). Cl-OPEs, including TCEP, TDCPP and TCIPP, are commonly used as plasticizers and flame retardants [56]. Among them, TCIPP is widely distributed in the environment because of its high production volume and its application in the production of plastics and PUFs [57]. Although there are limited reports of the presence of OPEs in soil samples, some studies have shown that their composition patterns in indoor dust and air are similar, where TCIPP also dominates and is detected with a higher frequency [46,58,59]. It is also the main OPE in household [60,61], office [62] and automobile [63] dust. Cl-OPE is also easily released from PUF products into the environment [37,64]. This is followed by Alkyl-OPEs, with the largest contributor being TBEP, which is the main OPE in the urban soils of Guangzhou and Chongqing, as well as in the Plastic Waste Disposal Area in Hebei Province, China [43,51,65]. In addition, the level of OPE-enriched soil in the Qinghai–Tibet Plateau is also high, and its main monomer is TBEP, which may be related to the local development of tourism and the abandonment of plastic waste by tourists and as a consequence of other human activities [49]. However, due to the uneven distribution of sampling points, the content of OPEs in Chinese soil cannot be fully represented. In addition, the data in some provinces are limited and may not accurately reflect the exposure of OPEs in the entire province.

#### 3.2.2. OPE Exposure Levels in Soils of Different Functional Zones

**Figure 3:**
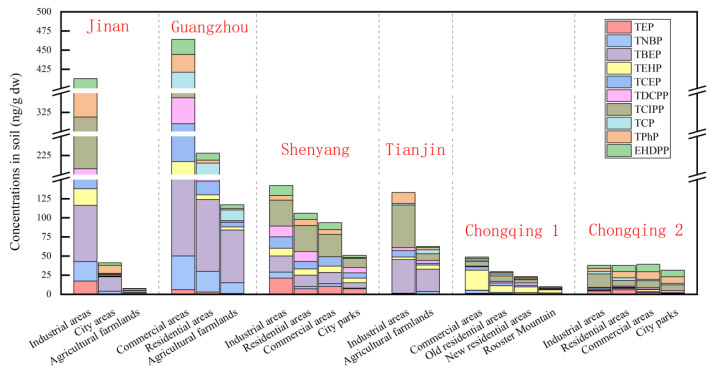
The occurrence level and composition of OPEs in the soils of different functional zones.

According to the type of land use, areas can be divided into different functional zones, mainly industrial areas, commercial areas, residential areas and agricultural lands. The concentration of ∑OPEs in soil in different functional areas was quite different. As shown in Figure 3, in the same area, the highest concentration of ∑OPEs is in the industrial area, and due to frequent industrial activities, OPEs are prone to enter the environment through volatilization and the discharge of effluents, both during and after industrial production. The mean value of ∑OPEs in the Jinan [25] industrial area reached 433 ng/g, which is fifty-five times higher than that of local agricultural soil (7.89 ng/g). Among them, TCIPP (111 ng/g) was dominant. This is followed by areas such as commercial and residential areas, where the consumption of OPEs is higher in large cities with a high population density and heavy traffic, due to the wide use of OPEs in vehicles, plastics, hydraulic fluids and lubricants [66], and because large quantities of building materials and commercial products may generate large quantities of organic pollutants and release them into the surrounding environment in a variety of ways [6]. According to the study, the average values of ∑OPEs in the soil of Guangzhou’s commercial and residential areas reached 460 ng/g and 230 ng/g, respectively [43], even higher than that of industrial areas in other regions. This shows that alongside industrial production, human activities are also a leading cause of pollution by OPEs. Relatively small concentrations are found in park soils and agricultural soils. Concentrations of ∑OPEs were higher in parks in Shenyang [47] (range: 53–140 ng/g) than in parks in Chongqing [53] (range: 10.7–70.5 ng/g), and it has also been shown that OPEs were detected in Central Park, which is farther away from the city (range: 10.7–70.5 ng/g). Due to lower degrees of human activity, long-distance migration via the atmosphere and sewage may be the main reasons for the enrichment of OPEs in these areas [20,43,67,68].

#### 3.2.3. OPE Exposure Levels in Soil at Different Times

**Figure 4:**
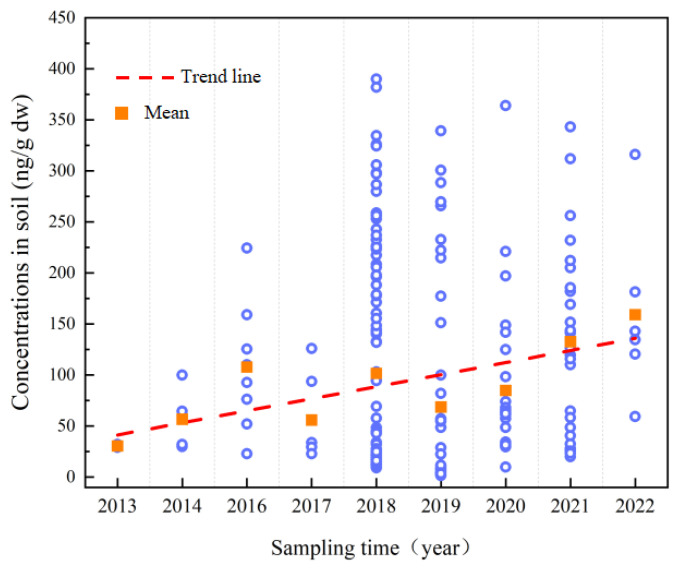
Temporal trend in OPE concentrations in soil from 2013 to 2022. The square represents the mean value of OPEs in samples from different years, and the dashed line represents the trend in the mean value.

Figure 4 shows the change in the concentration of OPEs in soil from 2013 to 2022. Our focus on and investigation of OPEs have gradually increased in recent years, significantly since 2018, when the detection and concentration of OPEs reported in studies increased significantly. Overall, the average concentration of OPEs in soil has increased from 30 ng/g in 2013 to 150 ng/g in 2022. As PBDEs are being phased out due to their environmental persistence and toxicity [69], OPEs are gradually becoming an alternative due to their good flame retardancy and physicochemical properties, and are widely used worldwide, with their global demand steadily increasing to approximately 1 million tons in 2018 [70]. China has become the largest producer and consumer of OPEs in the world, with its producers mainly located in east and central China [71].

### 3.3. Ecological Risks of OPEs

The widespread use of materials containing OPEs leads to their release into the soil in large quantities, posing a potential ecological risk. We obtained PNEC values for OPEs in soil based on toxicity data from terrestrial organisms. However, PNEC values for some OPEs, e.g., TEP, are unavailable due to a lack of toxicity data, so they are not subject to ecological risk assessment in this paper.

**Figure 5:**
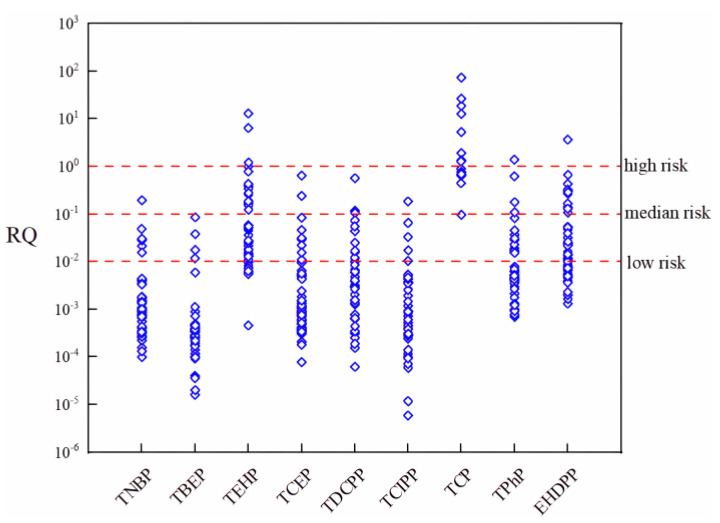
Risk quotient (RQ) of OPEs in soil samples. Dashed lines from top to bottom indicate RQ = 1, RQ = 0.1 and RQ = 0.01, respectively.

We carried out the risk assessment of OPEs in soils nationwide using the Risk quotient approach. The RQ values for ∑OPEs ranged from 5.9 × 10^−6^–72.5, with a mean value of 0.46 (Figure 5). Of the soil samples, 31.1% had 0.01 < RQ < 1, indicating low and medium risk. However, 3.2% of the samples had RQ > 1, indicating a high risk. Most of these samples were collected from soils in the industrial and commercial areas of economically developed regions. Among them, TBEP, TCIPP, TNBP, TCEP and TDCPP had RQ < 0.1 and at no point reached high risk, indicating that these OPEs pose a low risk to soil organisms. However, some OPEs had RQ values higher than 0.1, including TCP (mean: 9.06 ng/g), TEHP (mean: 0.51 ng/g) and EHDPP (mean: 0.14 ng/g), suggesting that these OPEs pose a greater ecological risk to soil organisms.

Similar results were reported for the soil survey (Table 1). The RQ of TEHP in Ningbo soil was >0.1, indicating medium and high risk [35,48]. The ecological risk assessment of parks in Beijing shows that for TCP, more than 24% of the points have RQ values between 0.1 and 1 [42]. However, due to the differences in the number of samples and the inconsistent land use of each sampling site, the risk level of some OPEs may be overestimated in the ecological risk assessment, and further research and verification are required.

## 4. Conclusions

This study statistically summarizes, for the first time, the exposure levels of ten common OPEs, including Cl-OPEs, Alkyl-OPEs and Aryl-OPEs, all new pollutants in our soils, and analyzes the source, toxicity and risk levels of OPEs. The results indicate that the detection level of OPEs in soil is generally higher in the country’s central-eastern, southern, and northeastern parts, such as Guangzhou province, Heilongjiang province and the city of Ningbo, which are generally economically developed or have a high degree of industrialization. Through the comparison of soil in different functional areas, it was found that the degree of OPE pollution in the soils of industrial and commercial areas is higher. Therefore, the management of OPEs in soils in highly urbanized cities needs to be strengthened. However, some remote areas, such as Tibet and Xinjiang, should also pay attention to OPE pollution brought on by the development of tourism. TCP, TBEP and TCIPP were found to be highly enriched in soil by analyzing the exposure levels of each OPEs. The overall level of OPE pollution has also increased in recent years. Meanwhile, this paper used the risk entropy method to assess the ecological risk of ten OPEs in soil samples across the country. The results indicate that TCP, TEHP and EHDPP pose high ecological risk, with most areas being at medium risk and above. These high-exposure and high-risk OPEs should be of great concern to society. Studies help people to understand the pollution and risk levels of OPEs. Consequently, China needs to pay attention to its governance and control measures in order to achieve sustainable development.

## Figures and Tables

**Table 1:** The concentration of ∑OPEs in soils across China.

Regions	∑OPEs	Median	Mean	Range	Analysis and Results	Reference
Helongjiang, Henan, Hubei, Guangxi	∑OPE11	229	230	62.3–394	OPEs are ubiquitous in farmland soils	[44]
Dalian	∑OPE8	56.5	58.5	33.1–136	The potential risk of TMPP (TCP) is medium to high	[45]
The Three Gorges Reservoir	∑OPE12	247	266	52.1–680	The concentrations of OPEs in riparian soil exceed those in farmland soil	[46]
Jinan	∑OPE10	34.9	106	2.55–581	Industrial soils have significantly higher levels of ΣOPE compared with those in urban and farmland soils	[25]
Shenyang	∑OPE13	156.2	229.8	38.7–952.2	OPE pollution levels are higher than farmland soils and lower than site soils	[47]
Ningbo	∑OPE7	——	469	163–986	TCIPP, TDCIPP, TBOEP and TEHP pose a medium potential risk	[48]
Qinghai–Tibet Plateau	∑OPE7	244	260.2	206.5–333.2	The main sources of OPEs in plateau soil are atmospheric wet and dry deposition, manufactured consumer materials and the release of OPEs from automobile interior decoration	[49]
Shanghai	∑OPE12	86.67	90.28	35.02–195.56	The difference in OPE pollution in different regions is great, showing the trend Shanghai > Xiuyan in Liaoning > Yanting in Sichuan > Xining in Qinghai	[50]
Shanghai	∑OPE12	78.58	78.58	40.54–173.29		
Sichuan	∑OPE12	22.26	22.35	10.46–35.65		
Sichuan	∑OPE12	28.55	36.82	5.46–127.87		
Qinghai	∑OPE12	16.97	17.82	4.47–40.2		
Qinghai	∑OPE12	28	30.03	9.22–46.9		
Liaoning	∑OPE12	17.13	18.34	3.4–32.87		
Liaoning	∑OPE12	36.22	49.62	9.77–195.54		
Chongqing	∑OPE10	61.2	77.4	10.1–315	The proportion of TBOEP(TBEP) is higher	[51]
Chongqing	∑OPE12	32.5	40.4	12–80.1	Exposure concentration is closely related to population density	[52]
Chongqing	∑OPE12	——	——	10.7–108	TCPP (TCIPP) and EHDPP are dominant compounds	[53]
Tibet	∑OPE9	62.01	50.8	29.74–73.87	Pollution mainly derived from building decoration materials, electronic products and polyurethane foam	[54]
China (farmland)	∑OPE11	4.9	——	2.41–35.8	The concentration of OPEs in soils in northeast and South China is significantly higher than that in northwest and central China	[21]
Beijing, Hebei, Tianjin	∑OPE12	3.914	6.72	0.543–54.9	Cl-OPEs are absolutely dominant, and TCIPP contributed the most	[22]
Guangzhou	∑OPE11	240	250	41–1300	On the whole, Guangzhou’s urban soil is moderately polluted by OPEs	[43]
Tianjin	∑OPE12	171	——	37.1–2700	Waste recycling is an important source of chlorinated- and aryl-OPFRs in the environment	[24]
Beijing	∑OPE11	157	299	21.4–2050	The ecological risk of OPEs in Beijing city park soil is at a medium level, and the risk of TCEP, TCIPP and TMPP(TCP) is relatively high	[42]

## Data Availability

Data are contained within the article.

## Supplementary Materials

The following supporting information can be downloaded at: https://www.mdpi.com/article/10.3390/toxics12090686/s1, Table S1: Physicochemical properties of OPEs selected in this study; Table S2: Summary of the concentrations of OPEs (ng/g dw) in the urban soils of China; Table S3: PNEC_soil_(ng/g) data on OPEs [72,73]. Figure S1: Spatial distribution characteristics and exposure levels of different OPEs.
