# Supplementary material for: Distribution Characteristics and Ecological Risk Assessment of Organophosphate Esters in Surface Soils of China
Source: Toxics. 2024 Sep 23;12(9):686. doi: 10.3390/toxics12090686 (PMC11435882; doi:10.3390/toxics12090686)
Supplement: Supplementary file 1 [file toxics-12-00686-s001.zip › toxics-3171826-supplementary.pdf]

Review

Distribution Characteristics and Ecological Risk Assessment of Organophosphate Esters in Surface Soils of China

Guorui Zhou, Yizhang Zhang, Ziye Wang, Mingrui Li, Haiming Li and Chen Shen

Table S1. Physicochemical properties of OPEs selected in this study.

Types	Abbreviation	Full name	Molecular formula	Molecular weight	CAS	Log K_{ow}	Log K_{oc}
Alkyl phosphate	TEP	Triethyl phosphate	$C_6H_{15}O_4P$	182.15	78-40-0	0.8	1.68
	TnBP	Tri-n-butyl phosphate	$C_{12}H_{27}O_4P$	266.31	126-73-8	4	3.27
	TBEP<br>（TBOEP）	Tributoxyethyl phosphate<br>Tris(1,3-dichloroisopropyl) phosphate	$C_{18}H_{39}O_7P$	398.47	78-51-3	3.65	5.66
	TEHP	Tris(2-ethylhexyl) phosphate	$C_{24}H_{51}O_4P$	434.63	78-42-2	9.49	6.87
Cl phosphate	TCEP	Tris(2-chloroethyl) phosphate	$C_6H_{12}Cl_3O_4P$	285.49	115-96-8	1.44	2.48
	TDCPP<br>（TDCIPP）	Tris(1,3-dichloro-2-propyl) phosphate<br>Tris(1,3-dichloroisopropyl) phosphate	$C_9H_{15}Cl_6O_4P$	430.91	13674-87-8	2.59	2.71
	TCIPP<br>（TCPP）	Tris(2-chloroisopropyl) phosphate	$C_9H_{18}Cl_3O_4P$	327.57	13674-84-5	2.89	3.10
Aryl phosphate	TCP<br>（TMPP）	Tricresyl phosphate<br>Tris(methylphenyl) phosphate	$C_{21}H_{21}O_4P$	368.36	1330-78-5	5.11	6.34
	TPhP	Triphenyl phosphate	$C_{18}H_{15}O_4P$	326.28	115-86-6	4.59	3.71
	EHDPP	2-Ethylhexyl diphenyl phosphate	$C_{20}H_{27}O_4P$	362.4	1241-94-7	6.3	4.2

Table S2. Summary of the concentrations of OPEs (ng/g dw) in urban soils of China.

Region	Chemicals	Median	Mean	Range	Functional areas	Sampling depth	Time
Anhui	TEP	0.14	0.31	N.D.—1.03	Agricultural farmlands	0-20cm	2019-2020
Beijing	TEP	0.17	0.18	0.05—0.31	Agricultural farmlands	0-20cm	2019-2020
Chongqing	TEP	4.5	5.7	N.D.—21.2	Multiple types	0-10cm	2016.7
Chongqing	TEP	——	0.77±0.19	——	University	0-10cm	2020.9
Chongqing	TEP	——	0.17±0.02	——	Rooster Mountain	0-10cm	2020.9
Chongqing	TEP	——	0.91±0.50	——	New residential areas	0-10cm	2020.9
Chongqing	TEP	——	0.64±0.90	——	Old residential areas	0-10cm	2020.9
Chongqing	TEP	——	1.17±1.11	——	Commercial areas	0-10cm	2020.9
Chongqing	TEP	——	5.92	——	Residential areas	0-10cm	2017.4
Chongqing	TEP	——	3.05	——	Industrial areas	0-10cm	2017.4
Chongqing	TEP	——	2.14	——	Commercial areas	0-10cm	2017.4
Chongqing	TEP	——	0.77	——	Park	0-10cm	2017.4
Chongqing	TEP	0.16	0.16	0.06—0.25	Agricultural farmlands	0-20cm	2019-2020
Four province	TEP	1.06	1.96	0.0399—26.4	Agricultural farmlands	0-20cm	2018.9-2019.3
Fujian	TEP	0.11	0.27	N.D.—1.8	Agricultural farmlands	0-20cm	2019-2020
Gansu	TEP	0.03	0.05	N.D.—0.18	Agricultural farmlands	0-20cm	2019-2020
Guangdong	TEP	0.04	0.06	N.D.—0.23	Agricultural farmlands	0-20cm	2019-2020
Guangxi	TEP	0.03	0.02	N.D.—0.15	Agricultural farmlands	0-20cm	2019-2020

Guangzhou	TEP	4	4	1.00—7.00	Agricultural farmlands	0-5cm	2011.12
Guangzhou	TEP	2	3	1—6	Residential areas	0-5cm	2011.12
Guangzhou	TEP	5	6	2—9	Commercial areas	0-5cm	2011.12
Guizhou	TEP	0.02	0.03	N.D.—0.14	Agricultural farmlands	0-20cm	2019-2020
Hainan	TEP	0.01	0.02	N.D.—0.13	Agricultural farmlands	0-20cm	2019-2020
Hebei	TEP	0.06	0.06	0.01—0.11	Agricultural farmlands	0-20cm	2019-2020
Heilongjiang	TEP	N.D.	N.D.	N.D.—0.03	Agricultural farmlands	0-20cm	2019-2020
Henan	TEP	0.25	0.25	N.D.—0.6	Agricultural farmlands	0-20cm	2019-2020
Hubei	TEP	0.1	3.17	N.D.—12.4	Agricultural farmlands	0-20cm	2019-2020
Hunan	TEP	0.29	2.7	N.D.—13.2	Agricultural farmlands	0-20cm	2019-2020
Inner Mongolia	TEP	0.08	0.07	N.D.—0.13	Agricultural farmlands	0-20cm	2019-2020
Jiangsu	TEP	0.01	0.13	N.D.—0.53	Agricultural farmlands	0-20cm	2019-2020
Jiangxi	TEP	N.D.	2.38	N.D.—13.4	Agricultural farmlands	0-20cm	2019-2020
Jilin	TEP	0.01	0.01	N.D.—0.04	Agricultural farmlands	0-20cm	2019-2020
Jinan	TEP	3.96	17.2	0.427—100	Industrial areas	0-10cm	2019.6
Jinan	TEP	0.128	0.165	<LOD—0.356	City areas	0-10cm	2019.6
Jinan	TEP	0.04	0.066	<LOD—0.181	Agricultural farmlands	0-10cm	2019.6
Liaoning	TEP	1.4	3.42	N.D.—9.42	Multiple types	0-20cm	2020.8
Liaoning	TEP	N.D.	0.12	N.D.—1.03	Multiple types	0-20cm	2021.1
Liaoning	TEP	0.01	0.04	N.D.—0.24	Agricultural farmlands	0-20cm	2019-2020
Ningxia	TEP	0.06	0.03	N.D.—0.12	Agricultural farmlands	0-20cm	2019-2020
Qinghai	TEP	10	8.67	N.D.—21.46	Multiple types	0-20cm	2020.8

Qinghai	TEP	13.55	16.23	N.D.—31.75	Multiple types	0-20cm	2021.1
Qinghai	TEP	0.1	0.14	0.04—0.37	Agricultural farmlands	0-20cm	2019-2020
Shaanxi	TEP	0.03	0.04	N.D.—0.12	Agricultural farmlands	0-20cm	2019-2020
Shandong	TEP	0.05	0.02	N.D.—0.16	Agricultural farmlands	0-20cm	2019-2020
Shanghai	TEP	0.22	1.36	N.D.—4.27	Multiple types	0-20cm	2020.8
Shanghai	TEP	8.18	8.24	4.05—13.07	Multiple types	0-20cm	2021.1
Shanghai	TEP	0.03	0.03	N.D.—0.06	Agricultural farmlands	0-20cm	2019-2020
Shanxi	TEP	0.05	0.05	0.01—0.1	Agricultural farmlands	0-20cm	2019-2020
Shenyang	TEP	5.1	10.2	＜u.d.l—52.1	City areas	0-10cm	2017.9
Sichuan	TEP	1.75	4.74	N.D.—13.87	Agricultural farmlands	0-20cm	2020.8
Sichuan	TEP	0.25	0.27	N.D.—0.94	Agricultural farmlands	0-20cm	2021.1
Sichuan	TEP	0.01	0.02	N.D.—0.06	Agricultural farmlands	0-20cm	2019-2020
Tianjin	TEP	0.07	0.07	0.02—0.16	Agricultural farmlands	0-20cm	2019-2020
Tianjin	TEP	0.42	0.55	＜MDL—1.34	Industrial areas	0-2cm	2017
Tianjin	TEP	0.42	0.38	N.D.—0.42	Agricultural farmlands	0-2cm	2017
Tibet	TEP	——	＜1	——	Multiple types	——	2020.8
Tibet	TEP	0.03	0.03	N.D.—0.1	Agricultural farmlands	0-20cm	2019-2020
Xinjiang	TEP	0.02	0.05	N.D.—0.14	Agricultural farmlands	0-20cm	2019-2020
Yunnan	TEP	0.07	0.09	0.02—0.22	Agricultural farmlands	0-20cm	2019-2020
Zhejiang	TEP	0.09	0.1	N.D.—0.3	Agricultural farmlands	0-20cm	2019-2020
Anhui	TNBP	0.87	0.97	0.04—2.72	Agricultural farmlands	0—20cm	2019—2020
Beijing	TNBP	3.21	3.01	2.19—3.62	Agricultural farmlands	0—20cm	2019—2020

Chongqing	TNBP	——	1.55±0.31	——	University	0—10cm	2020.9
Chongqing	TNBP	——	1.65±0.37	——	Rooster Mountain	0—10cm	2020.9
Chongqing	TNBP	——	1.40±0.72	——	Old residential areas	0—10cm	2020.9
Chongqing	TNBP	1.42	1.53	1.15—2.14	Agricultural farmlands	0—20cm	2019—2020
Chongqing	TNBP	——	3.98±4.79	——	Commercial areas	0—10cm	2020.9
Chongqing	TNBP	3	3.5	0.32—11.2	Multiple types	0—10cm	2016.7
Chongqing	TNBP	——	1.60±0.40	——	New residential areas	0—10cm	2020.9
Dalian	TNBP	17.5	19.4	8.39—41	Agricultural farmlands	0—10cm	2019.9
Four province	TNBP	0.249	0.246	＜LOD—1.96	Agricultural farmlands	0—20cm	2018.9—2019.3
Fujian	TNBP	1.31	1.2	0.21—1.69	Agricultural farmlands	0—20cm	2019—2020
Gansu	TNBP	N.D.	0.09	N.D.—0.73	Agricultural farmlands	0—20cm	2019—2020
Guangdong	TNBP	0.64	0.59	N.D.—1.65	Agricultural farmlands	0—20cm	2019—2020
Guangxi	TNBP	0.45	0.84	N.D.—3.26	Agricultural farmlands	0—20cm	2019—2020
Guangzhou	TNBP	14	14	2—25	Agricultural farmlands	0—5cm	2011.12
Guangzhou	TNBP	27	44	10—210	Commercial areas	0—5cm	2011.12
Guangzhou	TNBP	26	27	12—46	Residential areas	0—5cm	2011.12
Guizhou	TNBP	0.23	0.23	N.D.—0.55	Agricultural farmlands	0—20cm	2019—2020
Hainan	TNBP	0.34	0.62	0.04—1.91	Agricultural farmlands	0—20cm	2019—2020
Hebei	TNBP	0.02	0.14	N.D.—0.71	Agricultural farmlands	0—20cm	2019—2020
Heilongjiang	TNBP	0.49	0.5	N.D.—1.62	Agricultural farmlands	0—20cm	2019—2020
Henan	TNBP	0.53	0.72	N.D.—2.3	Agricultural farmlands	0—20cm	2019—2020
Hubei	TNBP	0.08	0.2	N.D.—1.19	Agricultural farmlands	0—20cm	2019—2020

Hunan	TNBP	0.29	0.36	N.D.—1.05	Agricultural farmlands	0—20cm	2019—2020
Inner Mongolia	TNBP	1.46	1.62	1.14—2.73	Agricultural farmlands	0—20cm	2019—2020
Jiangsu	TNBP	0.26	0.32	N.D.—0.73	Agricultural farmlands	0—20cm	2019—2020
Jiangxi	TNBP	0.13	0.28	N.D.—0.85	Agricultural farmlands	0—20cm	2019—2020
Jilin	TNBP	0.71	1.39	0.26—5.58	Agricultural farmlands	0—20cm	2019—2020
Jinan	TNBP	1.9	3.8	1.48—13.1	City areas	0—10cm	2019.6
Jinan	TNBP	8.15	25.5	<LOD — 83.0	Industrial areas	0—10cm	2019.6
Jinan	TNBP	0.783	0.693	<LOD—1.52	Agricultural farmlands	0—10cm	2019.6
Liaoning	TNBP	N.D.	0.84	N.D.—3.66	Multiple types	0—20cm	2020.8
Liaoning	TNBP	1.66	1.79	N.D.—4.69	Multiple types	0—20cm	2021.1
Liaoning	TNBP	0.38	0.12	N.D.—0.49	Agricultural farmlands	0—20cm	2019—2020
Ningbo	TNBP	——	5.24±5.07	1.28–33.5	Multiple types	0—20cm	2018.7
Ningxia	TNBP	0.32	0.62	N.D.—2.5	Agricultural farmlands	0—20cm	2019—2020
Qinghai	TNBP	0.21	0.21	N.D.—0.32	Multiple types	0—20cm	2020.8
Qinghai	TNBP	0.34	0.61	0.16—3.37	Multiple types	0—20cm	2021.1
Qinghai	TNBP	1.45	1.28	N.D.—1.96	Agricultural farmlands	0—20cm	2019—2020
Qinghai—Tibet Plateau	TNBP	——	2.5	0.6—5.8	Plateau region	0—10cm	2019.1
Shaanxi	TNBP	0.59	0.29	N.D.—1.41	Agricultural farmlands	0—20cm	2019—2020
Shandong	TNBP	0.2	0.39	N.D.—1.46	Agricultural farmlands	0—20cm	2019—2020
Shanghai	TNBP	1.28	1.23	N.D.—2.47	Agricultural farmlands	0—20cm	2019—2020
Shanghai	TNBP	N.D.	N.D.	N.D.	Multiple types	0—20cm	2020.8
Shanghai	TNBP	N.D.	0.84	N.D.—3.66	Multiple types	0—20cm	2021.1

Shanxi	TNBP	0.26	0.24	N.D.—0.55	Agricultural farmlands	0—20cm	2019—2020
Shenyang	TNBP	2.1	3.7	0.6—25.8	City areas	0—10cm	2017.9
Sichuan	TNBP	0.41	0.25	N.D.—0.65	Agricultural farmlands	0—20cm	2020.8
Sichuan	TNBP	0.27	0.33	0.16—0.65	Agricultural farmlands	0—20cm	2021.1
Sichuan	TNBP	0.1	0.28	N.D.—0.99	Agricultural farmlands	0—20cm	2019—2020
Tianjin	TNBP	3.05	3.07	＜MDL—4.68	Agricultural farmlands	0—2cm	2017
Tianjin	TNBP	1.09	0.78	N.D.—2.17	Industrial areas	0—2cm	2017
Tianjin	TNBP	0.69	0.9	N.D.—1.98	Agricultural farmlands	0—20cm	2019—2020
Tibet	TNBP	——	＜1	——	Multiple types	——	2020.8
Tibet	TNBP	0.26	0.31	N.D.—0.84	Agricultural farmlands	0—20cm	2019—2020
Xinjiang	TNBP	0.37	0.66	N.D.—2.13	Agricultural farmlands	0—20cm	2019—2020
Yunnan	TNBP	0.66	0.63	N.D.—1.44	Agricultural farmlands	0—20cm	2019—2020
Zhejiang	TNBP	0.12	0.3	N.D.—0.99	Agricultural farmlands	0—20cm	2019—2020
Anhui	TBEP/TBOEP	0.75	0.86	N.D.—1.92	Agricultural farmlands	0—20cm	2019—2020
Beijing	TBEP/TBOEP	0.92	1.2	N.D.—3.03	Agricultural farmlands	0—20cm	2019—2020
Chongqing	TBEP/TBOEP	——	0.43±0.40	——	University	0—10cm	2020.9
Chongqing	TBEP/TBOEP	——	N.D.	——	Rooster Mountain	0—10cm	2020.9
Chongqing	TBEP/TBOEP	——	0.94	——	Industrial areas	0—10cm	2017.4
Chongqing	TBEP/TBOEP	——	0.54	——	Park	0—10cm	2017.4
Chongqing	TBEP/TBOEP	——	0.10±0.18	——	Old residential areas	0—10cm	2020.9
Chongqing	TBEP/TBOEP	0.6	1.21	N.D.—3.39	Agricultural farmlands	0—20cm	2019—2020
Chongqing	TBEP/TBOEP	——	3.98±4.79	——	Commercial areas	0—10cm	2020.9

Chongqing	TBEP/TBOEP	——	1.14	——	Commercial areas	0—10cm	2017.4
Chongqing	TBEP/TBOEP	20.1	33.9	3.1—149	Multiple types	0—10cm	2016.7
Chongqing	TBEP/TBOEP	——	0.04±0.09	——	New residential areas	0—10cm	2020.9
Chongqing	TBEP/TBOEP	——	1.79	——	Residential areas	0—10cm	2017.4
Dalian	TBEP/TBOEP	17.9	19.49	4.66—61.3	Agricultural farmlands	0—10cm	2019.9
Four province	TBEP/TBOEP	0.367	0.434	0.210—1.05	Agricultural farmlands	0—20cm	2018.9—2019.3
Fujian	TBEP/TBOEP	0.73	1.18	N.D.—6.56	Agricultural farmlands	0—20cm	2019—2020
Gansu	TBEP/TBOEP	0.15	0.35	N.D.—1.59	Agricultural farmlands	0—20cm	2019—2020
Guangdong	TBEP/TBOEP	0.73	1.78	N.D.—8.69	Agricultural farmlands	0—20cm	2019—2020
Guangxi	TBEP/TBOEP	0.37	2.19	N.D.—13.4	Agricultural farmlands	0—20cm	2019—2020
Guangzhou	TBEP/TBOEP	68	69	17—114	Agricultural farmlands	0—5cm	2011.12
Guangzhou	TBEP/TBOEP	12	150	64—520	Commercial areas	0—5cm	2011.12
Guangzhou	TBEP/TBOEP	92	94	41—150	Residential areas	0—5cm	2011.12
Guizhou	TBEP/TBOEP	0.58	0.61	0.16—1.11	Agricultural farmlands	0—20cm	2019—2020
Hainan	TBEP/TBOEP	0.55	0.23	N.D.—0.99	Agricultural farmlands	0—20cm	2019—2020
Hebei	TBEP/TBOEP	0.65	0.29	N.D.—1.19	Agricultural farmlands	0—20cm	2019—2020
Heilongjiang	TBEP/TBOEP	0.44	0.67	N.D.—1.93	Agricultural farmlands	0—20cm	2019—2020
Henan	TBEP/TBOEP	0.59	0.71	N.D.—1.91	Agricultural farmlands	0—20cm	2019—2020
Hubei	TBEP/TBOEP	0.14	0.25	N.D.—0.96	Agricultural farmlands	0—20cm	2019—2020
Hunan	TBEP/TBOEP	0.01	1.19	N.D.—6.64	Agricultural farmlands	0—20cm	2019—2020
Inner Mongolia	TBEP/TBOEP	0.81	1.1	N.D.—2.94	Agricultural farmlands	0—20cm	2019—2020
Jiangsu	TBEP/TBOEP	0.82	0.93	0.28—2.12	Agricultural farmlands	0—20cm	2019—2020

Jiangxi	TBEP/TBOEP	0.13	2.77	N.D.—23.6	Agricultural farmlands	0—20cm	2019—2020
Jilin	TBEP/TBOEP	0.57	0.89	N.D.—2.85	Agricultural farmlands	0—20cm	2019—2020
Jinan	TBEP/TBOEP	11.3	18.8	0.923—49.7	City areas	0—10cm	2019.6
Jinan	TBEP/TBOEP	27.4	73.5	3.08—223	Industrial areas	0—10cm	2019.6
Jinan	TBEP/TBOEP	0.869	1.24	<LOD— 2.93	Agricultural farmlands	0—10cm	2019.6
Liaoning	TBEP/TBOEP	0	0.03	N.D.—0.61	Multiple types	0—20cm	2020.8
Liaoning	TBEP/TBOEP	0	1.24	N.D.—5.35	Multiple types	0—20cm	2021.1
Liaoning	TBEP/TBOEP	0.30.	0.05	N.D.—0.42	Agricultural farmlands	0—20cm	2019—2020
Ningbo	TBEP/TBOEP	——	330±154	64.8–896	Multiple types	0—20cm	2018.7
Ningxia	TBEP/TBOEP	0.72	0.83	N.D.—2.03	Agricultural farmlands	0—20cm	2019—2020
Qinghai	TBEP/TBOEP	N.D.	N.D.	N.D.	Multiple types	0—20cm	2020.8
Qinghai	TBEP/TBOEP	N.D.	N.D.	N.D.	Multiple types	0—20cm	2021.1
Qinghai	TBEP/TBOEP	0.34	0.48	N.D.—2	Agricultural farmlands	0—20cm	2019—2020
Qinghai—Tibet Plateau	TBEP/TBOEP	——	82.7	65.7—124.6	Plateau region	0—10cm	2019.1
Shaanxi	TBEP/TBOEP	0.01	0.64	N.D.—2.7	Agricultural farmlands	0—20cm	2019—2020
Shandong	TBEP/TBOEP	0.7	0.63	N.D.—1.15	Agricultural farmlands	0—20cm	2019—2020
Shanghai	TBEP/TBOEP	0.8	0.84	0.12—1.63	Agricultural farmlands	0—20cm	2019—2020
Shanghai	TBEP/TBOEP	N.D.	N.D.	N.D.	Multiple types	0—20cm	2020.8
Shanghai	TBEP/TBOEP	N.D.	0.52	N.D.—2.16	Multiple types	0—20cm	2021.1
Shanxi	TBEP/TBOEP	2	0.96	N.D.—5.17	Agricultural farmlands	0—20cm	2019—2020
Shenyang	TBEP/TBOEP	11.9	14.6	5.9—43.2	City areas	0—10cm	2017.9
Sichuan	TBEP/TBOEP	N.D.	N.D.	N.D.	Agricultural farmlands	0—20cm	2020.8

Sichuan	TBEP/TBOEP	N.D.	N.D.	N.D.	Agricultural farmlands	0—20cm	2021.1
Sichuan	TBEP/TBOEP	0.46	0.42	N.D.—1.03	Agricultural farmlands	0—20cm	2019—2020
Tianjin	TBEP/TBOEP	28.1	29.2	21.1—59.9	Agricultural farmlands	0—2cm	2017
Tianjin	TBEP/TBOEP	36.2	44	30.5—72.3	Industrial areas	0—2cm	2017
Tianjin	TBEP/TBOEP	0.07	0.98	N.D.—3.86	Agricultural farmlands	0—20cm	2019—2020
Tibet	TBEP/TBOEP	0.15	0.24	N.D.—0.87	Agricultural farmlands	0—20cm	2019—2020
Xinjiang	TBEP/TBOEP	0.46	1.1	N.D.—7.44	Agricultural farmlands	0—20cm	2019—2020
Yunnan	TBEP/TBOEP	0.61	0.7	0.02—1.52	Agricultural farmlands	0—20cm	2019—2020
Zhejiang	TBEP/TBOEP	0.49	0.87	0.18—2.24	Agricultural farmlands	0—20cm	2019—2020
Anhui	TEHP	0.13	0.29	N.D.—1.13	Agricultural farmlands	0—20cm	2019—2020
Beijing	TEHP	0.19	0.29	0.11—0.65	Agricultural farmlands	0—20cm	2019—2020
Chongqing	TEHP	——	46.1±20.7	——	University	0—10cm	2020.9
Chongqing	TEHP	——	4.80±0.20	——	Rooster Mountain	0—10cm	2020.9
Chongqing	TEHP	——	0.61	——	Industrial areas	0—10cm	2017.4
Chongqing	TEHP	——	0.28	——	Park	0—10cm	2017.4
Chongqing	TEHP	——	7.47±2.30	——	Old residential areas	0—10cm	2020.9
Chongqing	TEHP	0.33	0.47	0.13—1.38	Agricultural farmlands	0—20cm	2019—2020
Chongqing	TEHP	——	26.3±8.3	——	Commercial areas	0—10cm	2020.9
Chongqing	TEHP	——	2.7	——	Commercial areas	0—10cm	2017.4
Chongqing	TEHP	2.6	3.6	N.D.—13.3	Multiple types	0—10cm	2016.7
Chongqing	TEHP	——	8.60±2.31	——	New residential areas	0—10cm	2020.9
Chongqing	TEHP	——	0.54	——	Residential areas	0—10cm	2017.4

Four province	TEHP	143	140	3.10—210	Agricultural farmlands	0—20cm	2018.9—2019.3
Fujian	TEHP	0.74	1.01	0.07—2.93	Agricultural farmlands	0—20cm	2019—2020
Gansu	TEHP	0.11	1.08	N.D.—8.55	Agricultural farmlands	0—20cm	2019—2020
Guangdong	TEHP	0.15	0.31	N.D.—1.05	Agricultural farmlands	0—20cm	2019—2020
Guangxi	TEHP	0.16	1.27	N.D.—11.2	Agricultural farmlands	0—20cm	2019—2020
Guangzhou	TEHP	3	4	N.D.—15	Agricultural farmlands	0—5cm	2011.12
Guangzhou	TEHP	14	17	4—29	Commercial areas	0—5cm	2011.12
Guangzhou	TEHP	2	6	1—39	Residential areas	0—5cm	2011.12
Guizhou	TEHP	0.5	0.59	0.13—1.18	Agricultural farmlands	0—20cm	2019—2020
Hainan	TEHP	0.07	1.15	N.D.—7.31	Agricultural farmlands	0—20cm	2019—2020
Hebei	TEHP	0.03	0.24	N.D.—3.05	Agricultural farmlands	0—20cm	2019—2020
Heilongjiang	TEHP	0.26	0.38	0.03—1.18	Agricultural farmlands	0—20cm	2019—2020
Henan	TEHP	0.18	0.34	N.D.—1.67	Agricultural farmlands	0—20cm	2019—2020
Hubei	TEHP	0.12	0.16	N.D.—0.47	Agricultural farmlands	0—20cm	2019—2020
Hunan	TEHP	0.25	0.32	N.D.—0.95	Agricultural farmlands	0—20cm	2019—2020
Inner Mongolia	TEHP	0.07	0.4	N.D.—1.91	Agricultural farmlands	0—20cm	2019—2020
Jiangsu	TEHP	0.25	0.29	N.D.—0.64	Agricultural farmlands	0—20cm	2019—2020
Jiangxi	TEHP	0.28	0.32	0.17—0.53	Agricultural farmlands	0—20cm	2019—2020
Jilin	TEHP	0.08	0.18	N.D.—0.79	Agricultural farmlands	0—20cm	2019—2020
Jinan	TEHP	0.687	0.783	0.120—1.71	City areas	0—10cm	2019.6
Jinan	TEHP	1.03	21.7	0.815—100	Industrial areas	0—10cm	2019.6
Jinan	TEHP	0.212	0.212	<LOD— 0.528	Agricultural farmlands	0—10cm	2019.6

Liaoning	TEHP	0.23	2.96	N.D.—12.03	Multiple types	0—20cm	2020.8
Liaoning	TEHP	0.69	6.81	N.D.—63.85	Multiple types	0—20cm	2021.1
Liaoning	TEHP	0.11	0.18	N.D.—0.54	Agricultural farmlands	0—20cm	2019—2020
Ningbo	TEHP	——	14.1±13.0	3.51–93.1	Multiple types	0—20cm	2018.7
Ningxia	TEHP	0.06	0.01	N.D.—0.06	Agricultural farmlands	0—20cm	2019—2020
Qinghai	TEHP	N.D.	0.33	N.D.—4.45	Multiple types	0—20cm	2020.8
Qinghai	TEHP	N.D.	0.03	0.4	Multiple types	0—20cm	2021.1
Qinghai	TEHP	0.11	0.13	N.D.—0.34	Agricultural farmlands	0—20cm	2019—2020
Qinghai—Tibet Plateau	TEHP	——	4.4	3.9—13.4	Plateau region	0—10cm	2019.1
Shaanxi	TEHP	0.3	0.16	N.D.—1.05	Agricultural farmlands	0—20cm	2019—2020
Shandong	TEHP	0.28	0.47	N.D.—2.82	Agricultural farmlands	0—20cm	2019—2020
Shanghai	TEHP	0.53	0.58	N.D.—1.79	Agricultural farmlands	0—20cm	2019—2020
Shanghai	TEHP	5.34	7.54	2.15—22.59	Multiple types	0—20cm	2020.8
Shanghai	TEHP	6.44	9.83	4.20—38.97	Multiple types	0—20cm	2021.1
Shanxi	TEHP	0.01	0.01	N.D.—0.05	Agricultural farmlands	0—20cm	2019—2020
Shenyang	TEHP	7.2	8.4	＜u.d.l—22.1	City areas	0—10cm	2017.9
Sichuan	TEHP	3.31	2.87	0.50—4.34	Agricultural farmlands	0—20cm	2020.8
Sichuan	TEHP	9.13	12.73	0.53—44.27	Agricultural farmlands	0—20cm	2021.1
Sichuan	TEHP	0.08	0.29	N.D.—1.85	Agricultural farmlands	0—20cm	2019—2020
Tianjin	TEHP	1.61	5.62	1.26—45.7	Agricultural farmlands	0—2cm	2017
Tianjin	TEHP	1.78	3.33	0.93—12.0	Industrial areas	0—2cm	2017
Tianjin	TEHP	0.24	1.2	N.D.—8.48	Agricultural farmlands	0—20cm	2019—2020

Tibet	TEHP	2.74	2.74	——	Multiple types	——	2020.8
Tibet	TEHP	0.01	9.37	N.D.—92.64	Agricultural farmlands	0—20cm	2019—2020
Xinjiang	TEHP	0.69	0.12	N.D.—1.37	Agricultural farmlands	0—20cm	2019—2020
Yunnan	TEHP	0.32	0.39	N.D.—0.79	Agricultural farmlands	0—20cm	2019—2020
Zhejiang	TEHP	0.08	0.14	N.D.—0.43	Agricultural farmlands	0—20cm	2019—2020
Anhui	TCEP	0.35	0.08	N.D.—0.36	Agricultural farmlands	0—20cm	2019—2020
Beijing	TCEP	0.6	0.51	0.07—0.99	Agricultural farmlands	0—20cm	2019—2020
Chongqing	TCEP	——	3.80±1.49	——	University	0—10cm	2020.9
Chongqing	TCEP	——	0.71±0.03	——	Rooster Mountain	0—10cm	2020.9
Chongqing	TCEP	——	2.18	——	Industrial areas	0—10cm	2017.4
Chongqing	TCEP	——	0.29	——	Park	0—10cm	2017.4
Chongqing	TCEP	——	1.90±2.19	——	Old residential areas	0—10cm	2020.9
Chongqing	TCEP	0.09	0.03	N.D.—0.14	Agricultural farmlands	0—20cm	2019—2020
Chongqing	TCEP	——	4.26±5.40	——	Commercial areas	0—10cm	2020.9
Chongqing	TCEP	——	0.6	——	Commercial areas	0—10cm	2017.4
Chongqing	TCEP	10.2	11.3	N.D.—34.2	Multiple types	0—10cm	2016.7
Chongqing	TCEP	——	3.63±3.62	——	New residential areas	0—10cm	2020.9
Chongqing	TCEP	——	0.94	——	Residential areas	0—10cm	2017.4
Dalian	TCEP	3.69	3.87	0.63—8.72	Agricultural farmlands	0—10cm	2019.9
Four province	TCEP	17.9	32.3	1.63—152	Agricultural farmlands	0—20cm	2018.9—2019.3
Fujian	TCEP	0.4	0.45	N.D.—1.62	Agricultural farmlands	0—20cm	2019—2020
Gansu	TCEP	0.14	0.16	N.D.—0.44	Agricultural farmlands	0—20cm	2019—2020

Guangdong	TCEP	N.D.	0.07	N.D.—0.49	Agricultural farmlands	0—20cm	2019—2020
Guangxi	TCEP	0.24	0.43	N.D.—1.97	Agricultural farmlands	0—20cm	2019—2020
Guangzhou	TCEP	3	6	N.D.—47	Agricultural farmlands	0—5cm	2011.12
Guangzhou	TCEP	93	93	30—140	Commercial areas	0—5cm	2011.12
Guangzhou	TCEP	7	18	N.D.—110	Residential areas	0—5cm	2011.12
Guizhou	TCEP	0.29	0.22	N.D.—0.46	Agricultural farmlands	0—20cm	2019—2020
Hainan	TCEP	0.01	0.12	N.D.—0.47	Agricultural farmlands	0—20cm	2019—2020
Hebei	TCEP	0.09	0.14	N.D.—0.65	Agricultural farmlands	0—20cm	2019—2020
Heilongjiang	TCEP	N.D.	0.58	N.D.—5.69	Agricultural farmlands	0—20cm	2019—2020
Henan	TCEP	0.26	0.12	N.D.—0.58	Agricultural farmlands	0—20cm	2019—2020
Hubei	TCEP	0.21	0.27	N.D.—0.61	Agricultural farmlands	0—20cm	2019—2020
Hunan	TCEP	0.16	0.24	N.D.—1.18	Agricultural farmlands	0—20cm	2019—2020
Inner Mongolia	TCEP	0.38	0.4	N.D.—1.23	Agricultural farmlands	0—20cm	2019—2020
Jiangsu	TCEP	0.32	0.14	N.D.—0.6	Agricultural farmlands	0—20cm	2019—2020
Jiangxi	TCEP	0.41	0.42	0.18—0.83	Agricultural farmlands	0—20cm	2019—2020
Jilin	TCEP	0.88	0.32	N.D.—2.12	Agricultural farmlands	0—20cm	2019—2020
Jinan	TCEP	0.632	1.7	<LOD—4.64	City areas	0—10cm	2019.6
Jinan	TCEP	11.6	32.4	<LOD— 83.0	Industrial areas	0—10cm	2019.6
Jinan	TCEP	0.281	2.5	<LOD— 15.7	Agricultural farmlands	0—10cm	2019.6
Liaoning	TCEP	N.D.	N.D.	N.D.	Multiple types	0—20cm	2020.8
Liaoning	TCEP	7.86	12.04	N.D.—44.80	Multiple types	0—20cm	2021.1
Liaoning	TCEP	0.1	0.15	N.D.—0.6	Agricultural farmlands	0—20cm	2019—2020

Ningbo	TCEP	——	14.5±12.9	5.08–77.3	Multiple types	0—20cm	2018.7
Ningxia	TCEP	0.27	0.36	N.D.—1.12	Agricultural farmlands	0—20cm	2019—2020
Qinghai	TCEP	N.D.	N.D.	N.D.	Multiple types	0—20cm	2020.8
Qinghai	TCEP	N.D.	N.D.	N.D.	Multiple types	0—20cm	2021.1
Qinghai	TCEP	0.33	0.31	N.D.—0.8	Agricultural farmlands	0—20cm	2019—2020
Qinghai—Tibet Plateau	TCEP	——	16.9	11.6—31.9	Plateau region	0—10cm	2019.1
Shaanxi	TCEP	0.09	0.03	N.D.—0.11	Agricultural farmlands	0—20cm	2019—2020
Shandong	TCEP	0.64	0.19	N.D.—1.4	Agricultural farmlands	0—20cm	2019—2020
Shanghai	TCEP	0.46	1.67	N.D.—14	Agricultural farmlands	0—20cm	2019—2020
Shanghai	TCEP	N.D.	1.15	N.D.—4.77	Multiple types	0—20cm	2020.8
Shanghai	TCEP	1.71	6.49	N.D.—34.07	Multiple types	0—20cm	2021.1
Shanxi	TCEP	0.25	0.34	0.2—0.84	Agricultural farmlands	0—20cm	2019—2020
Shenyang	TCEP	7.6	12.1	＜u.d.l—56.1	City areas	0—10cm	2017.9
Sichuan	TCEP	1.92	1.76	N.D.—3.21	Agricultural farmlands	0—20cm	2020.8
Sichuan	TCEP	1.53	6.52	N.D.—27.84	Agricultural farmlands	0—20cm	2021.1
Sichuan	TCEP	0.07	0.12	N.D.—0.33	Agricultural farmlands	0—20cm	2019—2020
Tianjin	TCEP	1.72	2.03	1.72—3.79	Agricultural farmlands	0—2cm	2017
Tianjin	TCEP	6.39	8.52	＜MLD—23.0	Industrial areas	0—2cm	2017
Tianjin	TCEP	0.24	0.4	0.06—1.33	Agricultural farmlands	0—20cm	2019—2020
Tibet	TCEP	——	1.42	——	Multiple types	——	2020.8
Tibet	TCEP	0.12	0.2	N.D.—0.62	Agricultural farmlands	0—20cm	2019—2020
Xinjiang	TCEP	0.22	0.47	N.D.—2.77	Agricultural farmlands	0—20cm	2019—2020

Yunnan	TCEP	0.11	0.03	N.D.—0.19	Agricultural farmlands	0—20cm	2019—2020
Zhejiang	TCEP	0.08	0.13	N.D.—0.43	Agricultural farmlands	0—20cm	2019—2020
Anhui	TDCPP/TDCIPP	N.D.	0	N.D	Agricultural farmlands	0—20cm	2019—2020
Beijing	TDCPP/TDCIPP	N.D.	0.49	N.D.—2.34	Agricultural farmlands	0—20cm	2019—2020
Chongqing	TDCPP/TDCIPP	——	2.99±0.95	——	University	0—10cm	2020.9
Chongqing	TDCPP/TDCIPP	——	1.03±0.10	——	Rooster Mountain	0—10cm	2020.9
Chongqing	TDCPP/TDCIPP	——	0.86	——	Industrial areas	0—10cm	2017.4
Chongqing	TDCPP/TDCIPP	——	2.74	——	Park	0—10cm	2017.4
Chongqing	TDCPP/TDCIPP	——	4.29±1.40	——	Old residential areas	0—10cm	2020.9
Chongqing	TDCPP/TDCIPP	0.13	0.2	0.08—0.57	Agricultural farmlands	0—20cm	2019—2020
Chongqing	TDCPP/TDCIPP	——	6.20±5.60	——	Commercial areas	0—10cm	2020.9
Chongqing	TDCPP/TDCIPP	——	1.99	——	Commercial areas	0—10cm	2017.4
Chongqing	TDCPP/TDCIPP	3.2	5	N.D.—40.1	Multiple types	0—10cm	2016.7
Chongqing	TDCPP/TDCIPP	——	1.91±1.18	——	New residential areas	0—10cm	2020.9
Chongqing	TDCPP/TDCIPP	——	1.31	——	Residential areas	0—10cm	2017.4
Four province	TDCPP/TDCIPP	2.68	7.9	＜LOD—75.0	Agricultural farmlands	0—20cm	2018.9—2019.3
Fujian	TDCPP/TDCIPP	1.04	0.42	N.D.—3.1	Agricultural farmlands	0—20cm	2019—2020
Gansu	TDCPP/TDCIPP	0.67	0.1	N.D.—0.91	Agricultural farmlands	0—20cm	2019—2020
Guangdong	TDCPP/TDCIPP	1.26	0.25	N.D.—1.6	Agricultural farmlands	0—20cm	2019—2020
Guangxi	TDCPP/TDCIPP	1.37	0.25	N.D.—1.49	Agricultural farmlands	0—20cm	2019—2020
Guangzhou	TDCPP/TDCIPP	3	2	N.D.—11	Agricultural farmlands	0—5cm	2011.12
Guangzhou	TDCPP/TDCIPP	24	34	5—91	Commercial areas	0—5cm	2011.12

Guangzhou	TDCPP/TDCIPP	12	14	N.D.—50	Residential areas	0—5cm	2011.12
Guizhou	TDCPP/TDCIPP	1.53	1.52	N.D.—5.16	Agricultural farmlands	0—20cm	2019—2020
Hainan	TDCPP/TDCIPP	1.2	1.99	N.D.—5.35	Agricultural farmlands	0—20cm	2019—2020
Hebei	TDCPP/TDCIPP	1.35	0.21	N.D.—2.6	Agricultural farmlands	0—20cm	2019—2020
Heilongjiang	TDCPP/TDCIPP	N.D.	0.05	N.D.—0.64	Agricultural farmlands	0—20cm	2019—2020
Henan	TDCPP/TDCIPP	0.14	0.21	N.D.—2.1	Agricultural farmlands	0—20cm	2019—2020
Hubei	TDCPP/TDCIPP	5.23	5.38	0.43—12.6	Agricultural farmlands	0—20cm	2019—2020
Hunan	TDCPP/TDCIPP	0.57	0.41	N.D.—2.15	Agricultural farmlands	0—20cm	2019—2020
Inner Mongolia	TDCPP/TDCIPP	N.D.	0	N.D.—N.D.	Agricultural farmlands	0—20cm	2019—2020
Jiangsu	TDCPP/TDCIPP	N.D.	0	N.D.	Agricultural farmlands	0—20cm	2019—2020
Jiangxi	TDCPP/TDCIPP	0.42	2.15	N.D.—8.65	Agricultural farmlands	0—20cm	2019—2020
Jilin	TDCPP/TDCIPP	9.58	23.4	N.D.—99.6	Agricultural farmlands	0—20cm	2019—2020
Jinan	TDCPP/TDCIPP	0.773	0.968	<LOD —3.92	City areas	0—10cm	2019.6
Jinan	TDCPP/TDCIPP	12.7	37.7	0.319—102	Industrial areas	0—10cm	2019.6
Jinan	TDCPP/TDCIPP	0.127	0.209	<LOD— 0.775	Agricultural farmlands	0—10cm	2019.6
Liaoning	TDCPP/TDCIPP	4.55	5.73	1.77—11.17	Multiple types	0—20cm	2020.8
Liaoning	TDCPP/TDCIPP	10.3	10.43	N.D.—24.69	Multiple types	0—20cm	2021.1
Liaoning	TDCPP/TDCIPP	1.2	1.24	N.D.—3.27	Agricultural farmlands	0—20cm	2019—2020
Ningbo	TDCPP/TDCIPP	——	63.9±84.9	16.2–545	Multiple types	0—20cm	2018.7
Ningxia	TDCPP/TDCIPP	0.35	0.08	N.D.—0.69	Agricultural farmlands	0—20cm	2019—2020
Qinghai	TDCPP/TDCIPP	6.82	6.55	N.D.—15.10	Multiple types	0—20cm	2020.8
Qinghai	TDCPP/TDCIPP	6.9	7.03	5.29—10.50	Multiple types	0—20cm	2021.1

Qinghai	TDCPP/TDCIPP	0.01	0.02	N.D.—0.2	Agricultural farmlands	0—20cm	2019—2020
Qinghai—Tibet Plateau	TDCPP/TDCIPP	——	92.4	72.6—137.1	Plateau region	0—10cm	2019.1
Shaanxi	TDCPP/TDCIPP	0.33	0.06	N.D.—0.66	Agricultural farmlands	0—20cm	2019—2020
Shandong	TDCPP/TDCIPP	4.54	0.65	N.D.—7.77	Agricultural farmlands	0—20cm	2019—2020
Shanghai	TDCPP/TDCIPP	0.98	17.6	N.D.—157	Agricultural farmlands	0—20cm	2019—2020
Shanghai	TDCPP/TDCIPP	14.34	14.64	2.62—32.10	Multiple types	0—20cm	2020.8
Shanghai	TDCPP/TDCIPP	23.85	23.56	3.35—43.33	Multiple types	0—20cm	2021.1
Shanxi	TDCPP/TDCIPP	N.D.	0	N.D.—N.D.	Agricultural farmlands	0—20cm	2019—2020
Shenyang	TDCPP/TDCIPP	13.5	13.3	1.5—41	City areas	0—10cm	2017.9
Sichuan	TDCPP/TDCIPP	2.9	2.87	1.59—4.37	Agricultural farmlands	0—20cm	2020.8
Sichuan	TDCPP/TDCIPP	3.42	3.59	2.34—5.94	Agricultural farmlands	0—20cm	2021.1
Sichuan	TDCPP/TDCIPP	0.22	0.44	N.D.—2.78	Agricultural farmlands	0—20cm	2019—2020
Tianjin	TDCPP/TDCIPP	1.05	3.89	<MLD—28.3	Agricultural farmlands	0—2cm	2017
Tianjin	TDCPP/TDCIPP	29.1	3.78	<MLD—13.7	Industrial areas	0—2cm	2017
Tianjin	TDCPP/TDCIPP	0.52	0.11	N.D.—1.03	Agricultural farmlands	0—20cm	2019—2020
Tibet	TDCPP/TDCIPP	——	<1	——	Multiple types	——	2020.8
Tibet	TDCPP/TDCIPP	0.28	0.14	N.D.—0.89	Agricultural farmlands	0—20cm	2019—2020
Xinjiang	TDCPP/TDCIPP	N.D.	0	N.D.	Agricultural farmlands	0—20cm	2019—2020
Yunnan	TDCPP/TDCIPP	0.05	0.5	N.D.—2.02	Agricultural farmlands	0—20cm	2019—2020
Zhejiang	TDCPP/TDCIPP	N.D.	0	N.D.—N.D.	Agricultural farmlands	0—20cm	2019—2020
Anhui	TCIPP	0.13	0.51	N.D.—3.4	Agricultural farmlands	0—20cm	2019—2020
Beijing	TCIPP	0.45	1.04	0.2—3.45	Agricultural farmlands	0—20cm	2019—2020

Chongqing	TCIPP	——	1.51±0.21	——	University	0—10cm	2020.9
Chongqing	TCIPP	——	4.76±1.98	——	Rooster Mountain	0—10cm	2020.9
Chongqing	TCIPP	——	17.6	——	Industrial areas	0—10cm	2017.4
Chongqing	TCIPP	——	7.82	——	Park	0—10cm	2017.4
Chongqing	TCIPP	——	4.29±1.40	——	Old residential areas	0—10cm	2020.9
Chongqing	TCIPP	0.43	0.42	N.D.—0.86	Agricultural farmlands	0—20cm	2019—2020
Chongqing	TCIPP	——	6.20±5.60	——	Commercial areas	0—10cm	2020.9
Chongqing	TCIPP	——	9.08	——	Commercial areas	0—10cm	2017.4
Chongqing	TCIPP	1.3	3.3	N.D.—17.2	Multiple types	0—10cm	2016.7
Chongqing	TCIPP	——	7.27±3.64	——	New residential areas	0—10cm	2020.9
Chongqing	TCIPP	——	7.34	——	Residential areas	0—10cm	2017.4
Dalian	TCIPP	5.43	6.44	2.24—24.1	Agricultural farmlands	0—10cm	2019.9
Four province	TCIPP	3.59	4.42	0.948—16.4	Agricultural farmlands	0—20cm	2018.9—2019.3
Fujian	TCIPP	1.12	1.23	N.D.—3.03	Agricultural farmlands	0—20cm	2019—2020
Gansu	TCIPP	N.D.	0.17	N.D.—0.95	Agricultural farmlands	0—20cm	2019—2020
Guangdong	TCIPP	1.22	1.53	0.25—5.02	Agricultural farmlands	0—20cm	2019—2020
Guangxi	TCIPP	0.52	0.78	N.D.—1.86	Agricultural farmlands	0—20cm	2019—2020
Guangzhou	TCIPP	0.2	0.1	N.D.—1	Agricultural farmlands	0—5cm	2011.12
Guangzhou	TCIPP	3	6	1—16	Commercial areas	0—5cm	2011.12
Guangzhou	TCIPP	1	2	N.D.—14	Residential areas	0—5cm	2011.12
Guizhou	TCIPP	0.2	0.78	N.D.—5.09	Agricultural farmlands	0—20cm	2019—2020
Hainan	TCIPP	0.46	0.49	0.08—1.14	Agricultural farmlands	0—20cm	2019—2020

Hebei	TCIPP	0.03	0.16	N.D.—0.6	Agricultural farmlands	0—20cm	2019—2020
Heilongjiang	TCIPP	N.D.	0.41	N.D.—2.89	Agricultural farmlands	0—20cm	2019—2020
Henan	TCIPP	0.58	0.9	0.07—5.33	Agricultural farmlands	0—20cm	2019—2020
Hubei	TCIPP	0.74	1.57	0.33—5.65	Agricultural farmlands	0—20cm	2019—2020
Hunan	TCIPP	0.33	2.45	N.D.—24	Agricultural farmlands	0—20cm	2019—2020
Inner Mongolia	TCIPP	0.14	0.47	N.D.—1.78	Agricultural farmlands	0—20cm	2019—2020
Jiangsu	TCIPP	0.86	1.44	N.D.—4.43	Agricultural farmlands	0—20cm	2019—2020
Jiangxi	TCIPP	0.71	0.79	0.04—2.04	Agricultural farmlands	0—20cm	2019—2020
Jilin	TCIPP	0.94	1.52	N.D.—7.77	Agricultural farmlands	0—20cm	2019—2020
Jinan	TCIPP	0.136	1.04	<LOD —4.64	City areas	0—10cm	2019.6
Jinan	TCIPP	57	111	3.25—434	Industrial areas	0—10cm	2019.6
Jinan	TCIPP	<LOD	0.197	<LOD—1.63	Agricultural farmlands	0—10cm	2019.6
Liaoning	TCIPP	N.D.	0.38	N.D.—3.90	Multiple types	0—20cm	2020.8
Liaoning	TCIPP	0.79	7	N.D.—49.57	Multiple types	0—20cm	2021.1
Liaoning	TCIPP	0.55	0.69	N.D.—1.73	Agricultural farmlands	0—20cm	2019—2020
Ningbo	TCIPP	——	29.6±41.8	4.02–280	Multiple types	0—20cm	2018.7
Ningxia	TCIPP	0.07	0.01	N.D.—0.07	Agricultural farmlands	0—20cm	2019—2020
Qinghai	TCIPP	N.D.	N.D.	N.D.	Multiple types	0—20cm	2020.8
Qinghai	TCIPP	N.D.	N.D.	N.D.	Multiple types	0—20cm	2021.1
Qinghai	TCIPP	0.06	0.48	N.D.—1.91	Agricultural farmlands	0—20cm	2019—2020
Qinghai—Tibet Plateau	TCIPP	——	22	13.8—30.1	Plateau region	0—10cm	2019.1
Shaanxi	TCIPP	0.15	0.78	N.D.—5.48	Agricultural farmlands	0—20cm	2019—2020

Shandong	TCIPP	0.82	0.12	N.D.—1.08	Agricultural farmlands	0—20cm	2019—2020
Shanghai	TCIPP	N.D.	N.D.	N.D.—N.D.	Agricultural farmlands	0—20cm	2019—2020
Shanghai	TCIPP	N.D.	12.31	N.D.—69.97	Multiple types	0—20cm	2020.8
Shanghai	TCIPP	N.D.	N.D.	N.D.	Multiple types	0—20cm	2021.1
Shanxi	TCIPP	0.03	0.16	N.D.—0.79	Agricultural farmlands	0—20cm	2019—2020
Shenyang	TCIPP	15.3	29.4	1.3—209.3	City areas	0—10cm	2017.9
Sichuan	TCIPP	4.06	3.45	N.D.—8.61	Agricultural farmlands	0—20cm	2020.8
Sichuan	TCIPP	N.D.	0.38	N.D.—3.90	Agricultural farmlands	0—20cm	2021.1
Sichuan	TCIPP	1	1.04	N.D.—2.79	Agricultural farmlands	0—20cm	2019—2020
Tianjin	TCIPP	8.58	8.86	＜MLD—16.8	Agricultural farmlands	0—2cm	2017
Tianjin	TCIPP	29.1	55.7	5.39—176	Industrial areas	0—2cm	2017
Tianjin	TCIPP	0.31	0.54	N.D.—2.85	Agricultural farmlands	0—20cm	2019—2020
Tibet	TCIPP	——	＜1	N.D.—3.4	Multiple types	——	2020.8
Tibet	TCIPP	0.62	0.65	N.D.—1.37	Agricultural farmlands	0—20cm	2019—2020
Xinjiang	TCIPP	0.18	0.02	N.D.—0.18	Agricultural farmlands	0—20cm	2019—2020
Yunnan	TCIPP	0.25	0.52	N.D.—3.47	Agricultural farmlands	0—20cm	2019—2020
Zhejiang	TCIPP	0.2	0.24	N.D.—0.79	Agricultural farmlands	0—20cm	2019—2020
Chongqing	TCP/TMPP	——	1.35±0.37	——	University	0—10cm	2020.9
Chongqing	TCP/TMPP	——	0.26±0.04	——	Rooster Mountain	0—10cm	2020.9
Chongqing	TCP/TMPP	——	3.44	——	Industrial areas	0—10cm	2017.4
Chongqing	TCP/TMPP	——	1.87	——	Park	0—10cm	2017.4
Chongqing	TCP/TMPP	——	1.20±0.75	——	Old residential areas	0—10cm	2020.9

Chongqing	TCP/TMPP	——	1.73±2.48	——	Commercial areas	0—10cm	2020.9
Chongqing	TCP/TMPP	——	1.85	——	Commercial areas	0—10cm	2017.4
Chongqing	TCP/TMPP	——	2.41±5.24	——	New residential areas	0—10cm	2020.9
Chongqing	TCP/TMPP	——	3.63	——	Residential areas	0—10cm	2017.4
Four province	TCP/TMPP	19.9	34.4	＜LOD—130	Agricultural farmlands	0—20cm	2018.9—2019.3
Liaoning	TCP/TMPP	0	0.06	N.D.—1.18	Multiple types	0—20cm	2020.8
Liaoning	TCP/TMPP	N.D.	0.11	N.D.—2.16	Multiple types	0—20cm	2021.1
Qinghai	TCP/TMPP	N.D.	N.D.	N.D.	Multiple types	0—20cm	2020.8
Qinghai	TCP/TMPP	N.D.	N.D.	N.D.	Multiple types	0—20cm	2021.1
Shanghai	TCP/TMPP	6.04	6.76	N.D.—181.0	Multiple types	0—20cm	2020.8
Shanghai	TCP/TMPP	6.48	6.16	N.D.—13.56	Multiple types	0—20cm	2021.1
Sichuan	TCP/TMPP	N.D.	N.D.	N.D.	Agricultural farmlands	0—20cm	2020.8
Sichuan	TCP/TMPP	N.D.	0.24	N.D.—2.85	Agricultural farmlands	0—20cm	2021.1
Tibet	TCP/TMPP	——	＜1	——	Agricultural farmlands	0—20cm	2018.9—2019.3
Guangzhou	TCP/TMPP	13	14	N.D. —48	Agricultural farmlands	0—5cm	2011.12
Guangzhou	TCP/TMPP	28	71	9—450	Commercial areas	0—5cm	2011.12
Guangzhou	TCP/TMPP	46	51	20—110	Residential areas	0—5cm	2011.12
Anhui	TPhP	0.27	0.58	0.07—2.61	Agricultural farmlands	0—20cm	2019—2020
Beijing	TPhP	0.5	0.7	0.3—1.8	Agricultural farmlands	0—20cm	2019—2020
Chongqing	TPhP	——	3.78±2.62	——	University	0—10cm	2020.9
Chongqing	TPhP	——	0.28±0.05	——	Rooster Mountain	0—10cm	2020.9
Chongqing	TPhP	——	2.06±1.55	——	Old residential areas	0—10cm	2020.9

Chongqing	TPhP	0.4	0.58	0.3—1.61	Agricultural farmlands	0—20cm	2019—2020
Chongqing	TPhP	——	2.26±1.84	——	Commercial areas	0—10cm	2020.9
Chongqing	TPhP	3.2	4.8	N.D.—22.1	Multiple types	0—10cm	2020.9
Chongqing	TPhP	——	2.34±1.25	——	New residential areas	0—10cm	2020.9
Dalian	TPhP	——	2.49	0.5—26.4	Agricultural farmlands	0—10cm	2019.9
Four province	TPhP	3.14	3.76	＜LOD—153	Agricultural farmlands	0—10cm	2016.7
Fujian	TPhP	0.63	0.79	0.37—1.98	Agricultural farmlands	0—20cm	2019—2020
Gansu	TPhP	0.04	0.23	N.D.—1.39	Agricultural farmlands	0—20cm	2019—2020
Guangdong	TPhP	0.33	0.6	0.08—2.82	Agricultural farmlands	0—20cm	2019—2020
Guangxi	TPhP	0.2	0.55	N.D.—3.33	Agricultural farmlands	0—20cm	2019—2020
Guangzhou	TPhP	1	2	1—5	Agricultural farmlands	0—5cm	2011.12
Guangzhou	TPhP	21	23	5—46	Commercial areas	0—5cm	2011.12
Guangzhou	TPhP	4	4	2—9	Residential areas	0—5cm	2011.12
Guizhou	TPhP	0.1	0.39	0.02—1.45	Agricultural farmlands	0—20cm	2019—2020
Hainan	TPhP	0.5	0.51	0.08—1.19	Agricultural farmlands	0—20cm	2019—2020
Hebei	TPhP	0.01	0.13	N.D.—0.63	Agricultural farmlands	0—20cm	2019—2020
Heilongjiang	TPhP	0.75	1.02	0.11—3.03	Agricultural farmlands	0—20cm	2019—2020
Henan	TPhP	0.6	0.83	0.06—1.96	Agricultural farmlands	0—20cm	2019—2020
Hubei	TPhP	0.18	0.5	0.05—1.92	Agricultural farmlands	0—20cm	2019—2020
Hunan	TPhP	0.71	0.9	0.08—1.79	Agricultural farmlands	0—20cm	2019—2020
Inner Mongolia	TPhP	0.08	0.09	N.D.—0.21	Agricultural farmlands	0—20cm	2019—2020
Jiangsu	TPhP	0.48	0.57	0.2—1.18	Agricultural farmlands	0—20cm	2019—2020

Jiangxi	TPhP	0.29	0.54	0.11—2.66	Agricultural farmlands	0—20cm	2019—2020
Jilin	TPhP	0.22	0.68	N.D.—2.28	Agricultural farmlands	0—20cm	2019—2020
Jinan	TPhP	3.83	10.6	<LOD —47.6	City areas	0—10cm	2019.6
Jinan	TPhP	52.6	80.9	1.84—218	Industrial areas	0—10cm	2019.6
Jinan	TPhP	0.285	2.22	<LOD— 7.75	Agricultural farmlands	0—10cm	2019.6
Liaoning	TPhP	1.26	1.87	0.38—10.01	Multiple types	0—20cm	2020.8
Liaoning	TPhP	2.12	4.36	N.D.—40.53	Multiple types	0—20cm	2021.1
Liaoning	TPhP	0.35	0.61	0.07—2.28	Agricultural farmlands	0—20cm	2019—2020
Ningbo	TPhP	——	12.0±10.9	3.54–70.9	Multiple types	0—20cm	2018.7
Ningxia	TPhP	0.35	0.46	0.08—1.85	Agricultural farmlands	0—20cm	2019—2020
Qinghai	TPhP	0.43	0.47	N.D.—0.99	Multiple types	0—20cm	2020.8
Qinghai	TPhP	0.38	0.47	N.D.—1.24	Multiple types	0—20cm	2021.1
Qinghai	TPhP	0.03	0.1	N.D.—0.41	Agricultural farmlands	0—20cm	2019—2020
Qinghai—Tibet Plateau	TPhP	——	26.3	15.6—60.6	Plateau region	0—10cm	2019.1
Shaanxi	TPhP	0.26	0.35	N.D.—1.22	Agricultural farmlands	0—20cm	2019—2020
Shandong	TPhP	0.01	0.17	N.D.—1.84	Agricultural farmlands	0—20cm	2019—2020
Shanghai	TPhP	0.5	0.65	0.06—1.83	Agricultural farmlands	0—20cm	2019—2020
Shanghai	TPhP	1.16	1.31	0.74—2.52	Multiple types	0—20cm	2020.8
Shanghai	TPhP	1.9	2.21	1.11—3.94	Multiple types	0—20cm	2021.1
Shanxi	TPhP	0.06	0.17	N.D.—0.48	Agricultural farmlands	0—20cm	2019—2020
Shenyang	TPhP	2.6	5.9	0.3—79.5	City areas	0—10cm	2017.9
Sichuan	TPhP	0.21	0.28	N.D.—0.86	Agricultural farmlands	0—20cm	2020.8

Sichuan	TPhP	0.31	0.48	0.09—1.71	Agricultural farmlands	0—20cm	2021.1
Sichuan	TPhP	0.44	0.61	N.D.—1.33	Agricultural farmlands	0—20cm	2019—2020
Tianjin	TPhP	2.93	3	0.53—8.33	Agricultural farmlands	0—2cm	2017
Tianjin	TPhP	7.02	14.2	1.43—48.4	Industrial areas	0—2cm	2017
Tianjin	TPhP	0.48	0.55	0.07—0.99	Agricultural farmlands	0—20cm	2019—2020
Tibet	TPhP	——	1.01	——	Multiple types	——	2020.8
Tibet	TPhP	0.09	0.55	0.04—3.55	Agricultural farmlands	0—20cm	2019—2020
Xinjiang	TPhP	0.02	0.12	N.D.—0.72	Agricultural farmlands	0—20cm	2019—2020
Yunnan	TPhP	0.07	0.16	N.D.—0.6	Agricultural farmlands	0—20cm	2019—2020
Zhejiang	TPhP	0.45	0.66	0.1—1.58	Agricultural farmlands	0—20cm	2019—2020
Tianjin	EHDPP	1.23	1.57	1.12—4.58	Agricultural farmlands	0—2cm	2017
Anhui	EHDPP	0.03	0.05	N.D.—0.19	Agricultural farmlands	0—20cm	2019—2020
Beijing	EHDPP	0.19	0.49	N.D.—2.34	Agricultural farmlands	0—20cm	2019—2020
Chongqing	EHDPP	2.4	4.2	N.D.—13.2	University	0—10cm	2020.9
Chongqing	EHDPP	——	0.14±0.03	——	Rooster Mountain	0—10cm	2020.9
Chongqing	EHDPP	——	3.89	——	Industrial areas	0—10cm	2017.4
Chongqing	EHDPP	——	8.6	——	Park	0—10cm	2017.4
Chongqing	EHDPP	——	0.73±0.75	——	Old residential areas	0—10cm	2020.9
Chongqing	EHDPP	0.13	0.2	0.08—0.57	Agricultural farmlands	0—20cm	2019—2020
Chongqing	EHDPP	——	1.63±2.30	——	Commercial areas	0—10cm	2020.9
Chongqing	EHDPP	——	9.86	——	Commercial areas	0—10cm	2017.4
Chongqing	EHDPP	——	0.97±0.96	——	New residential areas	0—10cm	2020.9

Chongqing	EHDPP	——	8.06	——	Residential areas	0—10cm	2017.4
Dalian	EHDPP	1.45	1.44	0.35—2.92	Agricultural farmlands	0—10cm	2019.9
Four province	EHDPP	<LOD	<LOD	<LOD	Agricultural farmlands	0—20cm	2018.9—2019.3
Fujian	EHDPP	0.25	0.67	0.2—2.57	Agricultural farmlands	0—20cm	2019—2020
Gansu	EHDPP	0.11	0.17	0.02—0.48	Agricultural farmlands	0—20cm	2019—2020
Guangdong	EHDPP	0.22	0.34	0.1—1.51	Agricultural farmlands	0—20cm	2019—2020
Guangxi	EHDPP	0.37	1.19	0.16—6.62	Agricultural farmlands	0—20cm	2019—2020
Guangzhou	EHDPP	3	5	N.D.—22	Agricultural farmlands	0—5cm	2011.12
Guangzhou	EHDPP	18	20	6—40	Commercial areas	0—5cm	2011.12
Guangzhou	EHDPP	6	9	1—40	Residential areas	0—5cm	2011.12
Guizhou	EHDPP	0.2	0.26	N.D.—0.69	Agricultural farmlands	0—20cm	2019—2020
Hainan	EHDPP	0.1	0.16	0.03—0.56	Agricultural farmlands	0—20cm	2019—2020
Hebei	EHDPP	0.05	0.06	0.01—0.15	Agricultural farmlands	0—20cm	2019—2020
Heilongjiang	EHDPP	0.39	0.75	0.17—2.49	Agricultural farmlands	0—20cm	2019—2020
Henan	EHDPP	0.11	0.12	N.D.—0.33	Agricultural farmlands	0—20cm	2019—2020
Hubei	EHDPP	0.08	0.14	0.03—0.33	Agricultural farmlands	0—20cm	2019—2020
Hunan	EHDPP	0.2	0.24	N.D.—0.6	Agricultural farmlands	0—20cm	2019—2020
Inner Mongolia	EHDPP	0.28	0.3	0.01—0.79	Agricultural farmlands	0—20cm	2019—2020
Jiangsu	EHDPP	0.08	0.11	N.D.—0.38	Agricultural farmlands	0—20cm	2019—2020
Jiangxi	EHDPP	0.22	0.3	0.09—0.84	Agricultural farmlands	0—20cm	2019—2020
Jilin	EHDPP	0.13	0.42	0.06—2.55	Agricultural farmlands	0—20cm	2019—2020
Jinan	EHDPP	0.545	3.23	<LOD —11.7	City areas	0—10cm	2019.6

Jinan	EHDPP	0.589	13	<LOD —83.0	Industrial areas	0—10cm	2019.6
Jinan	EHDPP	0.207	0.36	<LOD—1.12	Agricultural farmlands	0—10cm	2019.6
Liaoning	EHDPP	0.51	0.72	N.D.—2.44	Multiple types	0—20cm	2020.8
Liaoning	EHDPP	0.62	0.92	N.D.—2.45	Multiple types	0—20cm	2021.1
Liaoning	EHDPP	0.2	0.43	0.03—1.33	Agricultural farmlands	0—20cm	2019—2020
Ningxia	EHDPP	0.15	0.15	0.06—0.27	Agricultural farmlands	0—20cm	2019—2020
Qinghai	EHDPP	N.D.	0.06	N.D.—0.50	Multiple types	0—20cm	2020.8
Qinghai	EHDPP	N.D.	0.03	0.37	Multiple types	0—20cm	2021.1
Qinghai	EHDPP	0.21	0.22	0.01—0.79	Agricultural farmlands	0—20cm	2019—2020
Shaanxi	EHDPP	0.17	0.15	N.D.—0.41	Agricultural farmlands	0—20cm	2019—2020
Shandong	EHDPP	0.07	0.11	N.D.—0.43	Agricultural farmlands	0—20cm	2019—2020
Shanghai	EHDPP	0.08	0.12	N.D.—0.32	Agricultural farmlands	0—20cm	2019—2020
Shanghai	EHDPP	0.35	15.07	0—130.64	Multiple types	0—20cm	2020.8
Shanghai	EHDPP	2.09	3.7	N.D.—11.03	Multiple types	0—20cm	2021.1
Shanxi	EHDPP	0.05	0.07	0.02—0.24	Agricultural farmlands	0—20cm	2019—2020
Shenyang	EHDPP	5.4	9.3	1.3—50	City areas	0—10cm	2017.9
Sichuan	EHDPP	N.D.	0.11	N.D.—0.33	Agricultural farmlands	0—20cm	2020.8
Sichuan	EHDPP	0.5	0.59	0.35—1.22	Agricultural farmlands	0—20cm	2021.1
Sichuan	EHDPP	0.11	0.11	0.03—0.15	Agricultural farmlands	0—20cm	2019—2020
Tianjin	EHDPP	0.18	0.04	0.17—0.81	Industrial areas	0—2cm	2017
Tianjin	EHDPP	0.22	0.66	0.03—2.22	Agricultural farmlands	0—20cm	2019—2020
Tibet	EHDPP	——	44.45	——	Multiple types	——	2020.8

Tibet	EHDPP	0.1	0.15	0.01—0.39	Agricultural farmlands	0—20cm	2019—2020
Xinjiang	EHDPP	0.08	0.79	0.01—5.43	Agricultural farmlands	0—20cm	2019—2020
Yunnan	EHDPP	0.1	0.43	0.02—2.34	Agricultural farmlands	0—20cm	2019—2020
Zhejiang	EHDPP	0.07	0.15	N.D.—0.48	Agricultural farmlands	0—20cm	2019—2020

*Four province: Heilongjiang, Henan, Hubei, Guangxi

Table S3. $PNEC_{soil}$（ng/g) data of OPEs.

OPEs	PNECsoil（ng/g）	Reference
TnBP	900	[1, 2]
TBEP（TBOEP）	2480	[1, 2]
TEHP	21.8	[1, 2]
TCEP	386	[3]
TDCPP（TDCIPP）	320	[3]
TCIPP（TCPP）	1700	[3]
TCP（TMPP）	2.7	[3]
TPhP	130	[3]
EHDPP	30.2	[3]

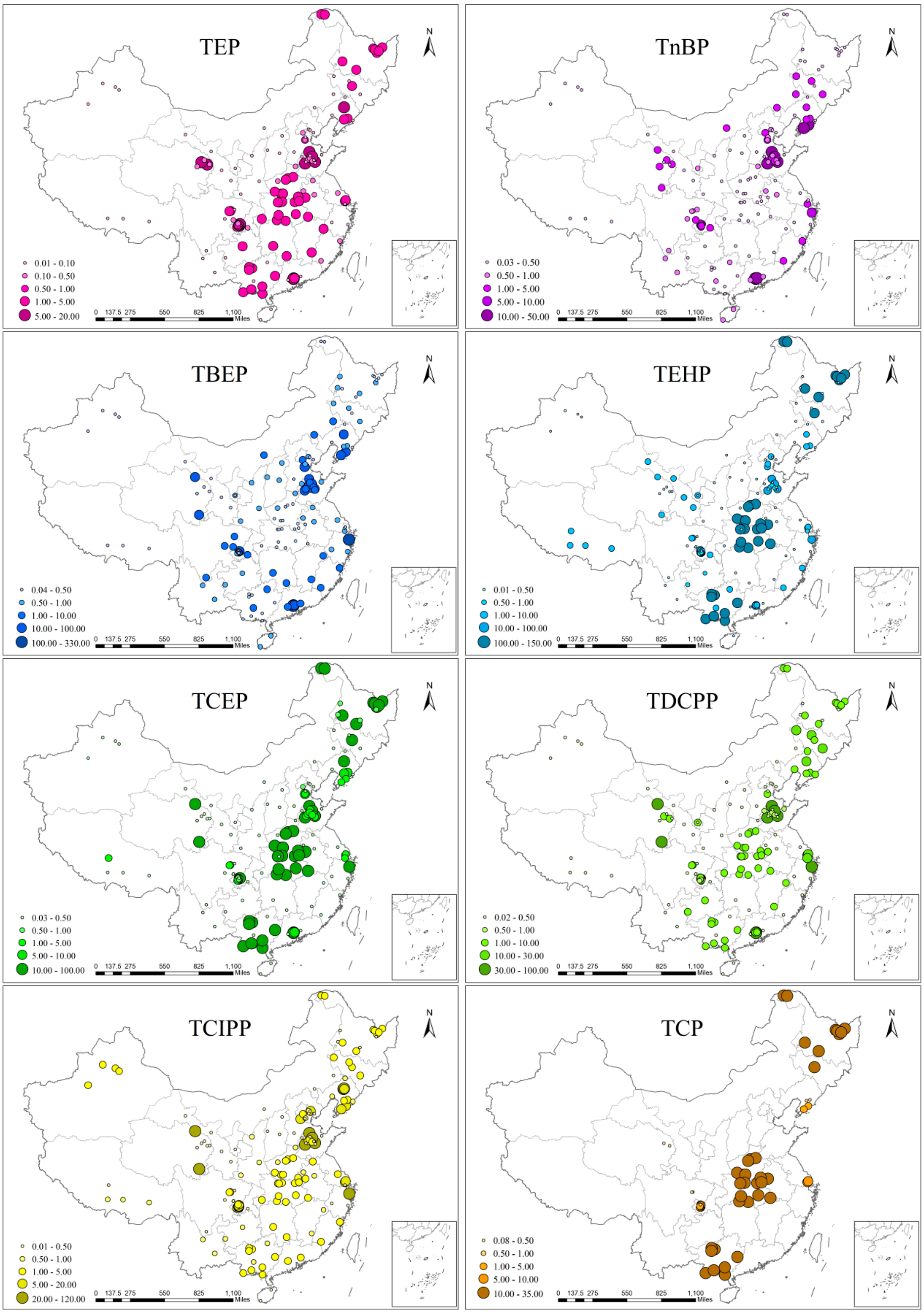
TEP
N
0.01 - 0.10
0.10 - 0.50
0.50 - 1.00
1.00 - 5.00
5.00 - 20.00
0 137.5 275 550 825 1,100 Miles
TnBP
N
0.03 - 0.50
0.50 - 1.00
1.00 - 5.00
5.00 - 10.00
10.00 - 50.00
0 137.5 275 550 825 1,100 Miles
TBEP
N
0.04 - 0.50
0.50 - 1.00
1.00 - 10.00
10.00 - 100.00
100.00 - 330.00
0 137.5 275 550 825 1,100 Miles
TEHP
N
0.01 - 0.50
0.50 - 1.00
1.00 - 10.00
10.00 - 100.00
100.00 - 150.00
0 137.5 275 550 825 1,100 Miles
TCEP
N
0.03 - 0.50
0.50 - 1.00
1.00 - 5.00
5.00 - 10.00
10.00 - 100.00
0 137.5 275 550 825 1,100 Miles
TDCPP
N
0.02 - 0.50
0.50 - 1.00
1.00 - 10.00
10.00 - 30.00
30.00 - 100.00
0 137.5 275 550 825 1,100 Miles
TCIPP
N
0.01 - 0.50
0.50 - 1.00
1.00 - 5.00
5.00 - 20.00
20.00 - 120.00
0 137.5 275 550 825 1,100 Miles
TCP
N
0.08 - 0.50
0.50 - 1.00
1.00 - 5.00
5.00 - 10.00
10.00 - 35.00
0 137.5 275 550 825 1,100 Miles

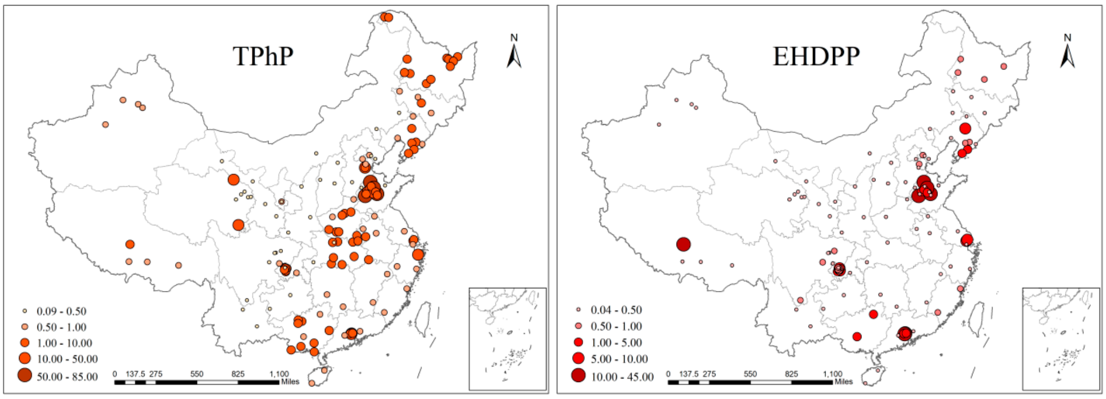

Figure. S1 Spatial distribution characteristics and exposure levels of different OPEs.

Reference:

1. U.S. Envrionmental Protection Agency. Exposure Assessment Tools and Models, Estimation Program Interface (EPI) Suite, V 4.1; U.S. Environmental Protection Agency, Exposure Assessment Branch: Washington, DC, 2011.
2. European Commission. Technical Guidance Document (TGD) on Risk Assessment of Chemical Substances (2nd edition). European Commission, European Chemical Bureau, Joint Research Centre, EUR 20418 EN/2. 2003.
3. European Commission. 2011. Identification and Evaluation of Data on Flame Retardants in Consumer Products. Contract 17.020200/09/ 549040.
